# AI‐Guided Intrinsic and Extrinsic Photocorrosion Inhibition of III–V Semiconductors for Durable Photoelectrochemical Water Splitting

**DOI:** 10.1002/smsc.70431

**Published:** 2026-10-29

**Authors:** Nafeesa Sarfraz, Ibrahim Khan, Muhammad Ibrar Ahmed, Muhammad Nawaz Tahir, Ajayan Vinu, Sanghyuk Wooh

**Affiliations:** ^1^ Department of Chemical Engineering Chung Ang University Seoul Republic of Korea; ^2^ Department of Chemical and Environmental Engineering Faculty of Science and Engineering University of Nottingham Ningbo China Ningbo China; ^3^ Global Innovative Center for Advanced Nanomaterials (GICAN) School of Science College of Engineering Science and Environment The University of Newcastle Callaghan New South Wales Australia; ^4^ Department of Chemistry College of Chemical Sciences King Fahd University of Petroleum and Minerals Dhahran Saudi Arabia; ^5^ Interdisciplinary Research Centre for Hydrogen Technologies and Carbon Management (IRC‐HTCM) King Fahd University of Petroleum & Minerals Dhahran Saudi Arabia

**Keywords:** artificial intelligence screening tools, corrosion inhibition, group III–V semiconductors, machine learning, photoelectrochemical water splitting, surface passivation

## Abstract

Group III–V semiconductors are suitable for solar‐driven hydrogen production due to precise lattice matching (for tandem device combinations), high carrier mobilities (>10^3^ cm^2^ V^–1^ s^–1^), tunable bandgaps (1.1–2.5 eV), strong absorption coefficients (>10^5^ cm^–1^), and a large range of doping possibilities. GaInP_2_, GaAs, and InP have a theoretical solar‐to‐hydrogen efficiency of more than 20%. Still, their high susceptibility to chemical and photochemical corrosion during prolonged photoelectrochemical water splitting (PEC‐WS) is a major industrial hurdle. This review comprehensively covers critical strategies to enhance the photostability of group III–V semiconductors for industrial‐scale PEC‐WS, emphasizing artificial intelligence (AI) and machine learning (ML) support. We discuss three main intrinsic and extrinsic strategies that include (i) AI‐driven crystal structure modification and faceted growth control, (ii) physical and chemical surface passivation coupled with machine learning for passivation material screening strategies, and (iii) combining III–V semiconductors with appropriate band‐aligned materials via preferable heterojunctions, utilizing algorithms to select optimal compositions. We examine the underlying mechanisms of each strategy and conclude with perspectives on achieving optimal materials for PEC‐WS using advanced AI and ML screening tools. Implementing these effective modification strategies, the wide commercial potential of III–V photoelectrodes for PEC‐WS technology can be unlocked.

## Graphical abstract

This article presents a comprehensive framework for the AI‐guided photocorrosion inhibition of III–V photoelectrodes. It explores how machine learning accelerates material discovery through targeted strategies in AI‐guided composition, passivation, and growth. Highlighting innovations in doping, morphological control, and protective capping, this data‐driven roadmap opens highly stable solar‐to‐hydrogen energy systems.

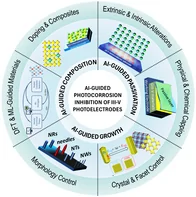

**Abbreviations**
*η*_HC_
: half‐cell efficiency
*η*_STH_
: solar to hydrogen efficiency
ABPE: applied‐bias photon‐to‐current efficiency
EQE: external quantum efficiency
FE: Faradaic efficiency
ff: fill factor
HVPE: Hydride vapor phase epitaxy
*I*_ox_
: oxidation current
*I*_ph_
: photocurrent
*I*_sc_
: saturated current
Kpi: potassium phosphate
MOCVD: metal‐organic chemical vapor deposition
PL: photoluminescence
*R*_S_
: series resistance
*V*_fb_
: flat band potential
*V*_oc_
: open circuit potential
*V*_ph_
: photo potential

## 1 Introduction

The development of a wide variety of high‐quality and low‐cost energy sources to meet the world's demand for energy without harming the environment is a major challenge [1]. Fossil fuels are currently responsible for 85% of the energy globally, and our increasing dependence on fossil fuels has accelerated the release of carbon dioxide (CO_2_) emissions, accelerating climate change and a public health crisis [2, 3, 4, 5]. According to the 2025 Global Carbon Budget, the trend is reinforced further as fossil fuels consumed in 2024 emitted more than 37 billion tons of CO_2_ (Figure 1a) [7]. The volatility and disruption in conventional energy markets, along with these climate realities, make the shift to clean energy alternatives imperative [8].

**FIGURE 1:**
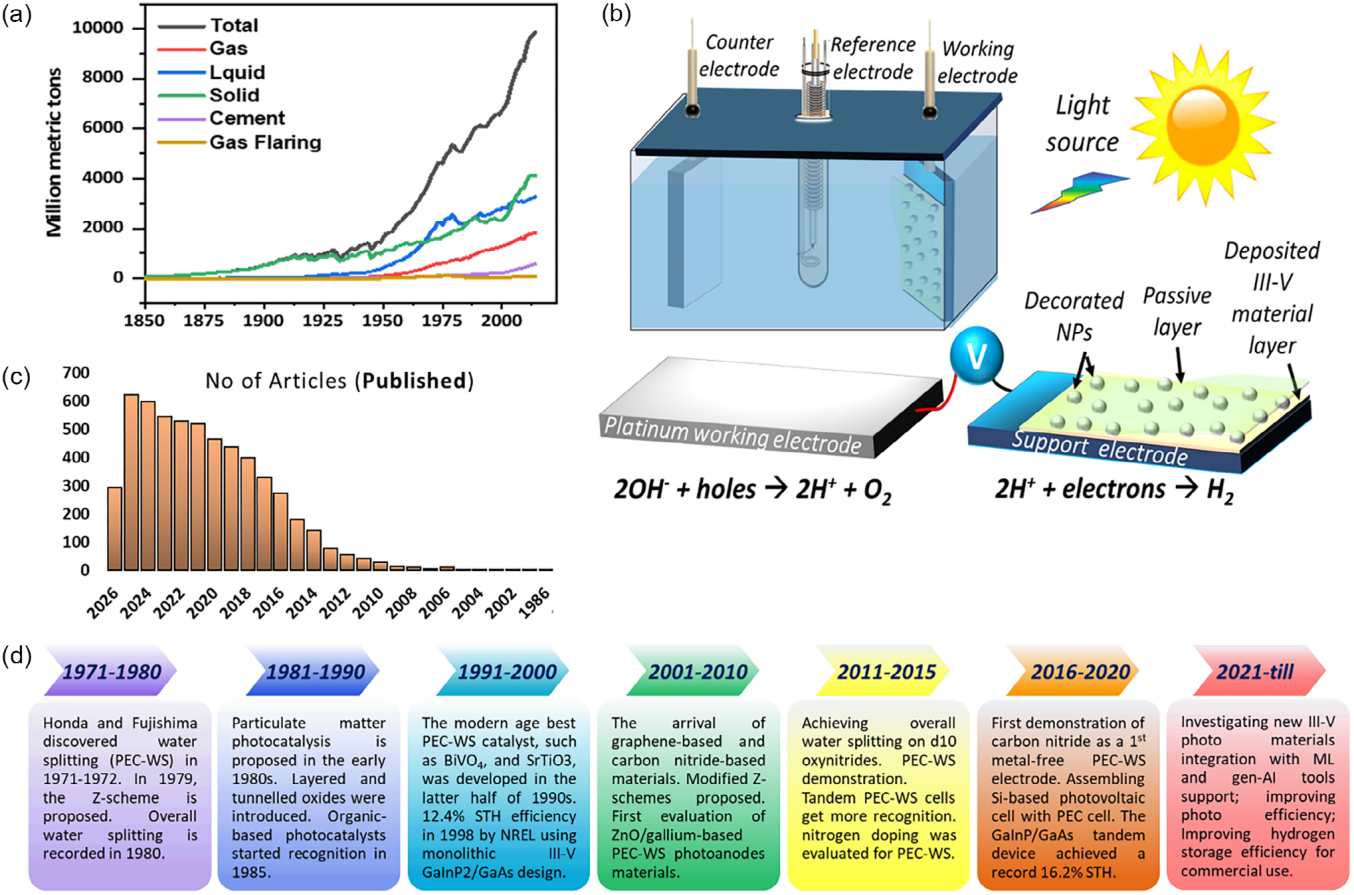
(a) Annual carbon emissions and individual contributors (1850–2025) from the CO_2_ Information Analysis Center (CDIAC) database (source: https://cdiac.ess‐dive.lbl.gov/trends/emis/glo_2014.html, accessed 15/03/2026). (b) Photoelectrochemical water splitting cell and mechanism over a surface‐passivated, nanoparticle‐decorated, and III–V‐deposited photoelectrode. (c) Annual publications on photoelectrochemical water splitting (2000–2026) from the Scopus database using the keyword “Photoelectrochemical water splitting; PEC water splitting” (accessed 24/04/2026). (d) Progress overview of photoelectrochemical water splitting materials and strategies, adapted with permission from ref. [6]. Copyright 2019, Elsevier.

Hydrogen, characterized by its high energy density and zero emissions, offers a promising clean energy solution [9, 10]. It is widely used in ammonia production through the Haber–Bosch process, glass purification, and petrochemical refining (desulfurization and gasoline reformulation), steel manufacturing, metallurgical processes, oxygen scavenging in heat treatment, and the hydrogenation of unsaturated fats and vegetable oils [11, 12, 13]. When the challenges of transportation and storage are addressed, hydrogen can also be relied upon for automobile (zero‐emission vehicle) fuel, as well as domestic energy. The European Union (EU) plan to become the world's first climate‐neutral continent by 2050 is underway following the adoption of the European Green Deal [14]. The development of clean energy through alternative technologies is an important area of research, especially hydrogen.

Conventional hydrogen production processes, such as steam methane reforming (SMR) and thermochemical processes, are still widely used in many sectors. In recent years, photocatalysis and electrocatalysis (PC & EC) have been a significant alternative with more economical and eco‐friendly attributes [15, 16, 17, 18, 19]. These multidisciplinary fields harness light and electrical energy to drive chemical reactions. Their integration marks a transition toward photoelectrochemical (PEC) technology, which is rapidly advancing due to its ability to generate clean fuel (hydrogen) from water splitting and convert CO_2_ into various valuable products [20, 21, 22, 23]. Compared with conventional catalytic methods, PEC technologies offer key advantages, including direct solar energy conversion and storage, and the facilitation of off‐grid and on‐demand energy systems [24]. While still in its early stage of development, it requires extensive research to meet performance benchmarks and become commercially viable. The pioneering work by Fujishima et al. on PEC water splitting (PEC‐WS) using wide‐bandgap TiO_2_ under ultraviolet (UV) light remains a classic model [25]. According to the literature, ideal PEC materials should (i) possess a bandgap of 1.5–2.0 eV, (ii) have optical band positions that straddle the hydrogen evolution reaction (HER) and oxygen evolution reaction (OER) water potential, (iii) efficiently absorb solar light, (iv) generate photoexcited carriers (electrons/holes), (v) minimize charge recombination and promote charge transport, and (vi) exhibit high solar‐to‐hydrogen (STH) and incident photon‐to‐current efficiency (IPCE) [26]. The conventional PEC‐WS mechanism involves three main steps (Figure 1b) that include (i) absorption of solar photons, (ii) generation and transport of charge carriers to reaction sites, and (iii) utilization of charge carriers for water splitting.

The effectiveness of PEC‐WS mainly depends upon the intrinsic and extrinsic characteristics of the working catalyzing materials [27, 28, 29, 30, 31]. The optimization of the physicochemical (surface, shape, size, crystal facets) and optoelectronic properties (band alignments, light absorption, plasmonic behavior) of PEC‐WS catalysts is a key research field. The utilization of electrochemically active co‐catalysts or surface passivation is considered effective for efficiency and stability enhancement [32, 33]. Over the past few years, different strategies have been employed to improve the PEC‐WS catalysts efficiency, which is reflected by the increasing number of annual publications ((Figure 1c) Scopus keyword search: photoelectrochemical water splitting; PEC water splitting).

Several materials, primarily TiO_2_, WO_3_, ZnO, BiVO_4_, Fe_2_O_3_, and CuO/Cu_2_O, have been explored for PEC‐WS [6, 8, 27, 34, 35, 36, 37, 38, 39, 40, 41]. Recently, photovoltaic active single‐crystal Si‐based architectures have shown potential for improved PEC performance. Due to their metastable nature, however, they experience crystal and stacking faults that make high‐quality growth challenging. This has resulted in the low experimental quantum efficiencies being less than expected. Furthermore, Si‐based materials tend to suffer from anodic corrosion under severe electrolytic conditions. Despite these issues, they exhibit promising PEC‐WS properties and warrant further investigation for practical application and commercialization [42, 43].

Group III–V semiconductors are promising alternatives to Si crystals due to unique intrinsic and extrinsic properties like lower direct bandgap, high light absorption, and excellent charge transport properties [44, 45, 46]. A III–V multijunction solar cell has already accomplished the feat of exceeding the power conversion efficiency (PCE) of silicon (Si) (30%) by ~1.5 times (47.1%) in solid‐state photovoltaics [47]. It is important to distinguish that this is a photovoltaic (PV) metric rather than a PEC STH efficiency. However, this record highlights the unparalleled light‐harvesting and carrier‐transport capabilities of III–V architectures, serving as a benchmark that inspires the translation of these materials into highly efficient PEC water‐splitting photoelectrodes. Several Group III–V semiconductors, such as GaAs, InP, GaInP_2_, and InAsP, have demonstrated significant PEC‐WS performance at the lab scale [48, 49, 50, 51]. However, their high costs, low stability in acid and chloride‐containing aqueous environments, recombination, and complex fabrication processes inhibit scalability and commercial application. Specifically, tuning the morphology (and other interfacial properties), cocatalysts, doping, and defects can conveniently adjust their optoelectronic properties. For example, a *p*‐type nanotextured InP photocathode from the III–V family achieved an exceptional photocurrent density of 37 mA cm^–2^ at 0.73 *V*_NHE_, with an STH of 14% [49]. For instance, an inverted metamorphic multijunction architecture based on GaInP/GaInAs tandem absorbers established a vital historical benchmark, achieving over 16% STH efficiency under advanced on‐sun benchmarking conditions [52]. Building upon this foundation, subsequent protected III–V tandem PEC devices have pushed peak efficiencies even higher. Notably, later work by Cheng et al. demonstrated 19.3% STH efficiency in an acidic electrolyte and 18.5% STH in a neutral electrolyte using a surface‐protected III–V tandem device [45]. Despite impressive photocurrent densities and STH efficiencies, their practical utilization in PEC‐WS is hindered by intrinsic thermodynamic instability, leading to corrosion in highly acidic electrolytes and under challenging electrochemical potentials. In addition to photocorrosion, developing the III–V semiconductors is inherently complex, time‐consuming, and expensive. Molecular beam epitaxy (MBE), metal‐organic chemical vapor deposition (MOCVD), plasma growth, and other related techniques are utilized for III–V semiconductor growth [53, 54, 55]. These techniques usually require costly precursors, multistep supervision, and care. A minor deviation in the growth recipe can lead to substantial financial losses. Based on the quality grade, a wafer costs 500–3000 $/wafer, much more than a silicon wafer [56].

Currently, there is limited literature addressing the use of artificial intelligence (AI) to fabricate III–V materials for optoelectronic applications [57, 58, 59, 60]. Nonetheless, to mitigate the photocorrosion that plagues Group III–V semiconductors in PEC‐WS, a multifaceted approach supported by machine learning (ML) and AI is highly critical [61, 62]. AI‐enhanced approaches can avoid crystal defects and accelerate optimal composition screening to design impactful, photocorrosion‐resistant III–V electrodes in a shorter period of time at a relatively lower cost. Importantly, at the molecular scale, the properties of III–V semiconductors depend on the exact arrangement of atoms and the degree of defects. AI‐supported machine learning tools can be helpful to determine precise molecular patterns during crystal growth [63, 64, 65, 66, 67]. In this scenario, machine learning potentials (MLPs) trained on relatively cheaper density functional theory (DFT) tools (such as the Perdew–Burke–Ernzerhof (PBE), generalized gradient approximation (GGA) functional) can perform atomic interactions simulations many times faster than conventional simulations [68]. Moreover, the real‐time crystal growth process optimization can be achieved with AI‐monitored reaction conditions to control molecular‐scale crystal lattice formation [69, 70]. Adaptive Bayesian optimization (AdBO) is a useful AI tool that can predict meticulous experimental conditions to achieve target crystallization kinetics. The AdBO guide can reduce material usage up to fivefold by screening the most appropriate experimental conditions [71].

The performance of photoelectrodes is more significantly affected by defects (like vacancies, dislocations, and antisites) at the molecular scale [72, 73, 74, 75, 76, 77]. Early‐stage identification during III–V wafer growth can help avoid additional growth steps and costs. Computer vision models, such as convolutional neural networks (CNNs), can be trained to analyze the morphological results from SEM, TEM, or AFM in an in situ fashion to detect the faults and adjust the growth conditions accordingly [78]. The AI‐driven molecular modeling and predictive simulation are found useful to decide optimal passivation layers, by theoretically predicting their electronic band alignments. Additionally, AI algorithms can swiftly analyze vast materials databases to identify novel, highly stable, and nontoxic materials for deposition. The implementation of AI‐powered digital twins for real‐time monitoring allows for the dynamic optimization of operating conditions such as pH, light intensity, and potential, to identify photostability “sweet spots” and predict failure points before they occur. Each of these strategies is supported by underlying AI tools to develop more authentic structures and significantly reduce the time and costs associated with experimental trial‐and‐error. Finally, AI can be extended to understand the atomic‐scale interfacial interactions and structures at electrochemical interfaces during PEC‐WS measurements. In this regard, Zhuang et al. introduced an artificial intelligence‐accelerated ab initio MD dataset called ElectroFace to visualize, compile, and provide open access to interface data, fostering collaboration and accelerating progress in electrochemistry [79]. Therefore, advancing III–V materials design with the support of ML tools will be key to better understanding interfacial properties, electrode/electrolyte interactions, and the effects of various physicochemical parameters on photostability.

This review provides an overview of existing ML‐ and AI‐assisted strategies for related systems and their possible implication for III–V PEC‐WS devices to achieve high hydrogen production performance. Three main strategies are discussed to address photocorrosion and photocurrent loss in III–V semiconductor electrodes: (i) AI‐driven crystal structure modifications and faceted growth control to optimize electrode/electrolyte interfacial properties to facilitate water molecule adsorption and H_2_ generation [80, 81]; (ii) physical and chemical surface passivation, incorporating machine learning‐supported passivation materials screening to promote the photostability of PEC devices under high pH and anodic conditions [82]; and (iii) combining III–V materials with appropriate bandgap materials via nanocomposite formation to minimize photoexcitons recombination via type‐I and type‐II heterostructure mechanisms, applying algorithms to select their suitable compositions [83, 84, 85]. Each strategy is supported by underlying ML and AI tools to develop more authentic structures at minimal production cost. Figure 1d shows the historical development of PEC‐WS technology and benchmark scenarios of III–V materials with a future perspective.

## 2 Intrinsic and Extrinsic Properties of III–V Semiconductors

### 2.1 Nanoscale Crystalline Orientations

Typically, the growth of III–V semiconductors follows two primary crystal phase orientations. Nitride compounds such as AlN, GaN, and AlGaN adopt wurtzite crystal structures (Figure 2a) with space group P63mc. In contrast, arsenides, antimonides, and phosphides of aluminum, gallium, and indium adopt a zinc blende crystal structure (Figure 2b) with space group F43m [86, 88, 89]. Table 1 summarizes the physicochemical parameters of these two phases.

**FIGURE 2:**
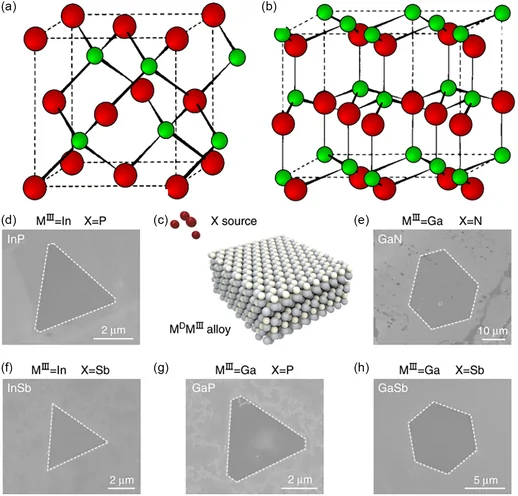
Representative unit cells (dashed lines) of (a) zinc blende (ZnS) and (b) wurtzite lattices (zinc and sulfur atoms are shown as green and red spheres, respectively). In III–V compounds, green spheres usually represent group III elements (Al, Ga, and In), while red spheres represent group V elements (N, P, As, and Sb). Reproduced with permission from ref. [86]. Copyright 2017, American Chemical Society. (c) Schematic of 2D‐LBL growth. (d–h) SEM of as‐fabricated InP, InSb, GaP, GaSb, and GaN single crystals, respectively. Reproduced from ref. [87] under the terms of the CC BY license.

**TABLE 1:** Group III–V compounds lattice and thermodynamic parameters measured at 298 K.

Crystal structure	Compound	Lattice parameter, Å	**Density, g cm** ^**−3**^	Melting point, K
Zinc blende	AIP	*a* = 5.46	2.40	3282
	AIAs	*a* = 5.67	3.76	2013
	AISb	*a* = 6.14	4.26	1338
	GaP	*a* = 5.45	4.14	1740
	GaAs	*a* = 5.56	5.32	1511
	InP	*a* = 5.87	4.81	1335
	InAs	*a* = 6.06	5.67	1215
	InSb	*a* = 6.48	5.77	798
Wurtzite	AIN	*a* = 3.11, *c* = 4.98	3.26	3273
	GaN	*a* = 3.19, *c* = 5.19	6.15	2773
	InN	*a* = 3.54, *c* = 5.70	6.81	1373

Besides these conventional orientations, single‐crystal and ultra‐thin III–V semiconductors are also highly intriguing. Currently, their synthesis is in its infancy, as the intrinsically strong covalent bonding in three‐dimensional (3D) orientations hinders the layer‐by‐layer (LBL) exfoliation during anisotropic growth. This issue was addressed by Chen et al., who grew various ultra‐thin, single III–V crystals by constructing an appropriate alloy substrate with enhanced adhesion strength. They determined that the material–substrate interaction plays a key role in the LBL growth of the ultra‐thin III–V crystals. They have successfully achieved the 2D LBL growth of InP, InSb, GaP, GaSb, and GaN III–V single crystals with specific atomic orientations as shown in Figure 2c–h [87].

### 2.2 Optoelectrical Characteristics at Nanoscale‐Absorption Coefficient

The optoelectronic properties of any photocatalytic materials are associated with space group (growth‐direction dimension), morphology, exposed surface area, particle size, crystal structure/exposed facets, and direct/indirect bandgap energies [90, 91]. Thus, it is essential to comprehend the intrinsic optical behavior of III–V semiconductors. Binary III–V semiconductors such as GaAs (1.42 eV), InAs (0.35 eV), GaP (2.3 eV), InP (1.34 eV), GaSb (0.73 eV), and InSb (0.17 eV) have been explored for PEC‐WS applications. GaAs, GaSb, GaP, and InP utilize the visible‐mid IR region, while InAs and InSb are sensitive to far‐IR radiation. It is possible to tune the bandgap energies through the introduction of extrinsic crystal defects, control of the dopant impurity concentration, and adjusting morphological changes. For example, in ultrathin 2D GaN III–V semiconductors, their optical properties are usually blue‐shifted compared with their bulk counterparts due to the monolayer structure. Similarly, the 2D optical absorption peak of InN shifts significantly toward the near‐infrared region [92]. This shift is also observed in 2D InP and InSb‐based III–V semiconductors [93]. Quantum confinement in these materials can be tuned by adjusting thickness and particle size to modify their bandgap for UV applications. Optical properties also depend on the ratio of alloying constituents. For instance, reducing Ga vacancies in monolayer GaN increases the bandgap, thus altering the optoelectrical behavior [94].

Moreover, piezoelectric behavior is influenced by layer number and morphology, which can further impact the optoelectrical behavior of the material. III–V semiconductors with hexagonal planar and buckled structures exhibit piezoelectricity owing to broken inversion symmetry [95]. Their anomalous thermoelectric properties are another feature. For instance, monolayer GaN has an analogous structure to graphene. However, its thermal conductivity is much lower. The phonon–phonon scattering selection rule is violated due to crystal symmetry distortion caused by the different atomic radius and mass of Ga and N atoms. Furthermore, lower thermal conductivity is associated with the localized charge density and strong Ga─N bond polarization, making it highly useful in broad energy conversion applications [92, 96].

Even though a few intrinsic properties are tunable and advantageous, several key challenges still need to be addressed before the full optoelectronic potential of nanoscale III–V materials can be realized in commercial applications. The 2D and faceted III–V structures have an extremely high surface‐to‐volume ratio, accelerating surface oxidation and photocorrosion under extreme anodic potentials [97, 98]. Also, a series of processes for developing strain‐free, defect‐free single crystals at the wafer scale is very costly and technically demanding. In the future, generative AI and machine learning can be used to overcome these challenges. Utilizing predictive computational models to swiftly screen vast compositional spaces for novel ternary and quaternary III–V alloy candidates with optimal band alignments and acceptable thermodynamic stability will shift the paradigm of material discovery from costly trial‐and‐error to rational design.

### 2.3 Mechanistic Origins and Challenges of III–V Photocorrosion

A rigorous understanding of photocorrosion mechanisms is essential for interpreting stability data and designing durable PEC‐WS devices [99]. The degradation of III–V semiconductors in aqueous electrolytes under illumination is a complex phenomenon governed by the interplay of thermodynamic driving forces, kinetic barriers, and operational parameters that collectively determine whether a material corrodes or performs stable water splitting [46, 51, 100]. To design robust protection strategies, it is necessary to explicitly distinguish between three mechanistically different contributions to degradation. These include (i) pre‐existing surface or growth defects acting as recombination centers, (ii) electrolyte‐induced photocorrosion or dissolution on an initially well‐defined surface, and (iii) new defect states generated during operation as a consequence of ongoing corrosion.

The fundamental thermodynamic and chemical instability of the semiconductor‐electrolyte junction is the primary durability problem, leading to degradation even on high‐quality, defect‐free epitaxial III–V surfaces. For most III–V semiconductors, the thermodynamic oxidation and reduction potentials in aqueous solution reveal that these materials are thermodynamically unstable under water‐splitting conditions, especially under anodic bias [100, 101, 102]. The valence band edge position is strongly coupled to the thermodynamic stability, setting fundamental limits for the highest achievable stability. However, kinetic stabilization through catalytic surface layers, protective coatings, or favorable reaction pathways can dramatically slow corrosion rates even when the overall reaction is thermodynamically downhill. The presence of an efficient electrocatalyst such as Pt or Rh can kinetically favor the desired water‐splitting reaction over the competing photocorrosion pathway by providing a lower‐overpotential route for charge transfer, and this kinetic competition between water oxidation or reduction and semiconductor decomposition is central to understanding device stability [103, 104]. The decomposition potential of a semiconductor is not a fixed material property but depends on crystal orientation, doping density, and defect structure [37].

For n‐type GaAs, InP, GaN, etc., III–V photoanodes, photocorrosion is anticipated when the decomposition potential lies below the valence band edge position, and experimentally, photocurrent at voltages negative of the water‐oxidation thermodynamic potential is primarily indicative of electrochemical photocorrosion rather than productive water splitting [105]. Regarding pre‐existing imperfections (Mechanism i), dislocations on GaAs surfaces, for example, lead to a more negative microscopic decomposition potential, making these sites particularly susceptible to corrosive attack. This local variation also explains why corrosion often initiates at specific surface features and propagates from these nucleation sites [106, 107]. However, even in the absence of pre‐existing defects, electrolyte‐induced photocorrosion occurs on initially pristine surfaces (Mechanism ii). Photocorrosion is fundamentally a charge‐carrier‐driven process; under illumination, photogenerated holes can oxidize the semiconductor directly for photoanodes or photogenerated electrons can reduce it for photocathodes, competing with the desired water‐splitting reactions. Potential cycling to values beyond 1.5 V_RHE_ leads to high anodic currents, rapid and visible pitting and etching of the semiconductor surface, and corresponding irreversible decreases in the light‐limited current density. For p‐type III–V photocathodes, photogenerated electrons drive the hydrogen evolution reaction but can also induce reductive corrosion. The outer protective layer of the III–V photocathode can be dissolved shortly after exposing it to PEC‐WS stability tests in the absence of a good RHE mediator such as Pt or Rh. For instance, a p‐doped GaAs/Si photocathode modified with sulfur passivation showed excellent photostability of 112 h upon interfacing with a Pt catalyst [108].

While sometimes mistaken for good passivating layers, uncontrolled native oxides often trigger photocorrosion and dissolution of III–V photoelectrodes under light due to imbalanced surface energies. Therefore, managing the electrode interface is critical for high‐performance PEC‐WS. The nanoporous, density‐graded surface of “black” gallium indium phosphide (GaInP_2_) was polished to remove the native oxide and then treated with (NH_4_)_2_S surface passivation to achieve a lifetime over 124 h via strongly suppressed corrosion kinetics [51]. Mechanistically, controlling native oxides in III–V compounds is challenging due to the multiple species involved. For example, the interface of GaAs can compete for two native oxides, Ga_2_O_3_ and As_2_O_3_, and volatile arsenic species can leave behind elemental arsenic clusters at the interface. The native oxide that can develop in InP can be InPO_4_ or In(PO_3_)_3_ and, under certain conditions, forms a bilayer to passivate the interface. However, the experimental conditions must be carefully controlled. The oxide layers are dynamic and critical when the material is operating. In aqueous electrolyte, oxides can dissolve. In the presence of light, both holes and electrons migrate and perform electrochemical reactions. This can expose fresh semiconductor surfaces, which in turn can lead to corrosion. Oxide growth and dissolution dictate the corrosive or protective regime in a loop reactor.

This dynamic degradation leads directly to Mechanism (iii): defect states generated during operation. Surface reconstruction is another dynamic issue that is associated with prolonged III–V photoelectrode tests for PEC‐WS and could cause severe photocorrosion. During long‐term PEC‐WS, the surface atoms rearrange, driving the leaching of Group V anions (e.g., P, As, Sb) into the electrolyte. The corrosion caused by reconstruction starts with the overaccumulation of leached oxide species. The reconstructed layer typically forms highly disordered, nonpassive oxides (as discussed above). In contrast to steady passivating films, the oxidized layers that are reconstructed are porous, allowing water molecules to constantly infiltrate the deeper regions of the underlying bulk semiconductor and dissolve it [109]. Furthermore, this surface reconstruction breaks the periodicity of the bulk lattice, generating off‐periodicity structural defects such as dangling bonds or broken dimers. These rearrangements of atoms cause the generation of mid‐gap electronic states, and these traps carriers. Therefore, it is vital to recognize that surface passivation (neutralizing pre‐existing electronic defects), corrosion protection (preventing chemical dissolution), and operational stability are highly related but not equivalent concepts.

Figure 3a illustrates how these contributions combine in a real protected device, using the degradation cascade identified by Suresh Babu et al. for a Pt/TiO_2_/III–V multijunction photocathode operated under unassisted water splitting conditions [110]. Front‐contact potential measurements, nanoscale imaging, and elemental analysis of both the surface and the electrolyte showed that degradation is initiated by detachment of the Pt catalyst. This results from poor wetting and weak adhesion of Pt on the TiO_2_ protection layer (steps I and II). Loss of Pt raises the overpotential required for the HER and shifts the local surface potential to more negative values, which drives TiO_2_ into a thermodynamically unstable region of its Pourbaix diagram and leads to dissolution of the protection layer (step III). The exposed AlInP window layer then dissolves, and the GaInP top junction undergoes cathodic corrosion with release of Ga^3+^, metallic In, and PH_3_ (step IV). The GaAs junction is exposed next and degrades with release of Ga^3+^ and AsH_3_ (step V), and finally the Ge bottom junction dissolves (step VI). The same sequence was observed in two‐ and three‐electrode configurations, which shows that the local catalyst potential rather than the externally applied bias governs the corrosion. This case study demonstrates that the initiator of failure was interfacial adhesion rather than a pre‐existing lattice defect. However, the porous oxides and mid‐gap states discussed above act as accelerants once the protection layer has been breached. Interface engineering that improves catalyst adhesion is therefore a prerequisite for durable III–V photocathodes, and stability tests should track the catalyst loading and the front‐contact potential together with the photocurrent.

**FIGURE 3:**
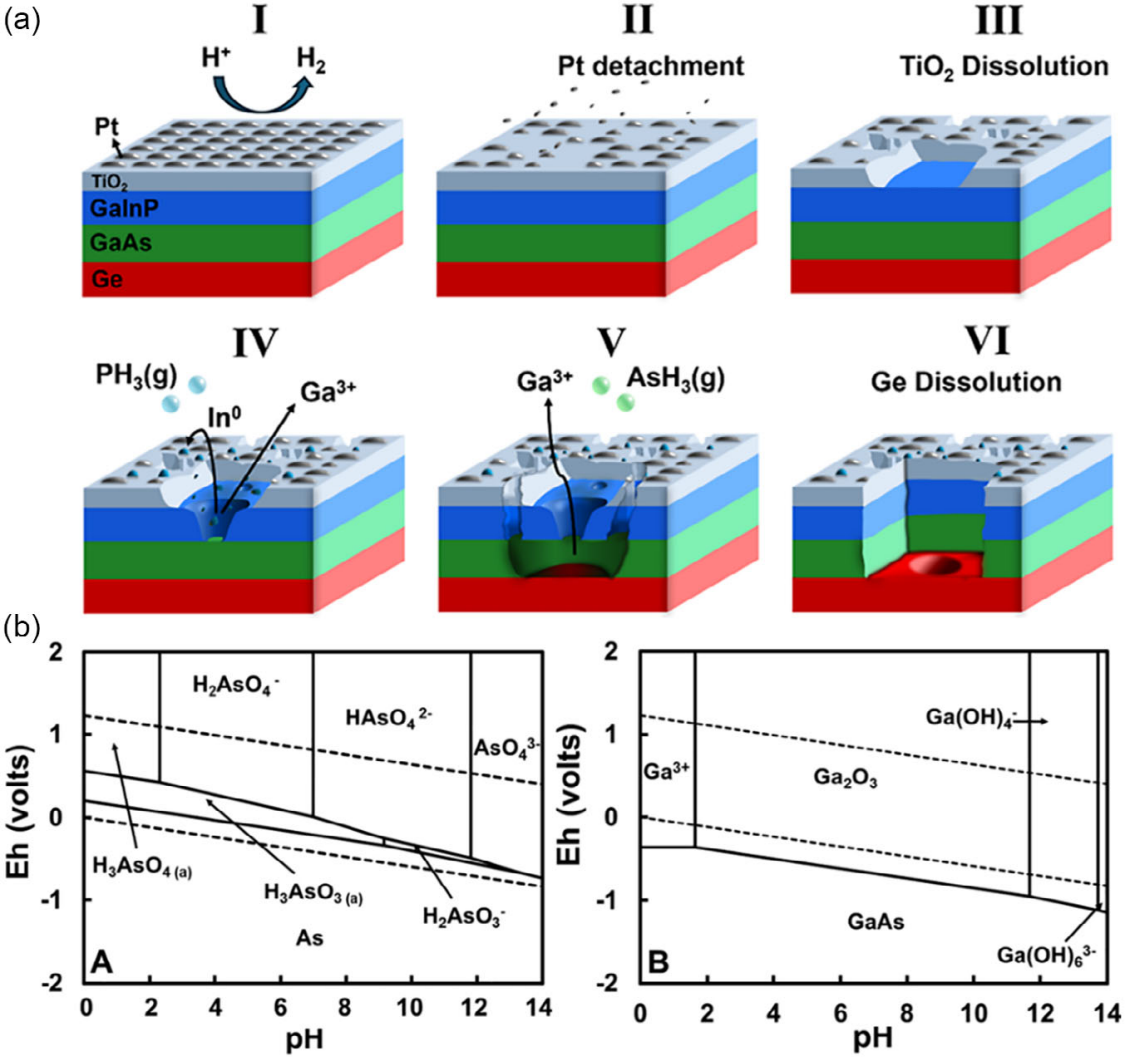
(a) Sequential degradation cascade of a Pt/TiO_2_‐protected GaInP/GaAs/Ge multijunction photocathode during unassisted water splitting. (I) Hydrogen evolution on the intact Pt/TiO_2_ surface. (II) Detachment of Pt particles caused by poor wetting and adhesion of Pt on TiO_2_. (III) Dissolution of the TiO_2_ protection layer after the local surface potential has shifted to more negative values. (IV) Cathodic corrosion of the exposed GaInP top junction with release of Ga^3+^, In^0^ and PH_3_(g). (V) Degradation of the GaAs junction with release of Ga^3+^ and AsH_3_(g). (VI) Dissolution of the Ge bottom junction. The thin AlInP window layer between TiO_2_ and GaInP is not drawn separately. Adapted with permission from ref. [110] Copyright 2025, American Chemical Society. (b) Pourbaix (potential versus pH) diagrams for the Ga–As–H_2_O system (6.5 mM As and 6.5 mM Ga) showing the predominant As species (left panel) and Ga species (right panel) over the full pH range. Dashed lines mark the stability window of water. Reproduced with permission from ref. [111] Copyright 2018, Elsevier.

The photocorrosion behavior of III–V semiconductors is highly sensitive to electrolyte pH as well. Different pH environments induce distinct element dissolution pathways and corrosion morphology evolution. Historically, GaAs has been shown to exhibit self‐passivation in pH 3–9 electrolytes during water oxidation. A potential/pH diagram (Pourbaix diagram) for the GaAs‐H_2_O system was drawn to identify the thermodynamically stable Ga and As species in aqueous systems (Figure 3b). In Pourbaix diagrams, the dashed lines mark the stability region of water. According to Figure 3b, As is soluble in the entire pH range. Arsenous acid (H_3_AsO_3_) is the only stable species at low redox potentials; meanwhile, ionic forms of arsenic acid (H_2_AsO_4_^−^ and HAsO_4_^2−^) are the stable As species in the pH range of 6.7–8.5 [111]. In contrast, in the same pH range, GaAs oxidized to form a native Ga_2_O_3,_ which is poorly soluble in water. The soluble Ga species, Ga^3+^, exists at pH < 1.6, and Ga(OH)_4_ is stable at pH > 13.7. These trends suggest that GaAs would form a surface layer of Ga_2_O_3_, with As dissolving in solution. Figure 3b shows that the upper stability limits of GaAs (−0.358 and −1.146 V at pH 0 and pH 14, respectively) are slightly lower than those of water, which indicates that GaAs is thermodynamically unstable in aqueous solution within the whole pH range.

Accumulated reaction products, electrolyte impurities, or degraded species on the surface of an electrode may lead to a higher series resistance, blocking of active sites, and increased recombination. The performance of a junction can be impaired by other mechanisms unrelated to photocorrosion, such as changes in doping profiles, defect migration, or interfacial reactions, whether or not they result in a change in band alignment. Therefore, rigorous stability studies must employ multiple characterization techniques to distinguish between these degradation pathways. Electrochemical impedance spectroscopy (EIS), combined with analysis of the distribution of relaxation times, can provide insight into the processes underpinning degradation. These techniques can be further complemented by chemical analysis, such as inductively coupled plasma mass spectrometry (ICP‐MS) of the electrolyte composition, and microscopic imaging, such as optical interferometry, to enhance the correlation between impedance response and material dissolution. Only through such comprehensive characterization can the true origin of performance loss be identified and appropriate mitigation strategies be developed.

## 3 III–V Materials Synthesis and Their Growth Mechanism

The design of III–V semiconductors having controlled optoelectrical properties has been done by different techniques. Epitaxial growth, for example, helps in depositing III–V semiconductor layers several micrometers or nanometers thick on monocrystalline substrates. In MOCVD (metal organic chemical vapor deposition method), sufficiently heated selective carrier transport gases (like H_2_, N_2_, Ar, He, etc.) are controlled to transport metal organic precursors in vapor phase onto the heated substrate to form a crystalline III–V semiconductor layer (Figure 4a) [86]. While MOCVD is versatile, it is complex, expensive, and potentially hazardous due to the use of multiple reagents and chemical reactions [115, 116]. Hot filament chemical vapor deposition (HF‐CVD) is another method for synthesizing III–V semiconductors. Gaseous precursor molecules disintegrate on a filament (which acts like a hot catalyst surface) to produce free radicals, which form a layer on the substrate surface. By controlling substrate and filament temperatures, filament composition, filament/substrate gap, and vacuum pressure, product quality can be improved [117, 118]. The HF‐CVD technique is scalable and applicable to both rigid and flexible substrates.

**FIGURE 4:**
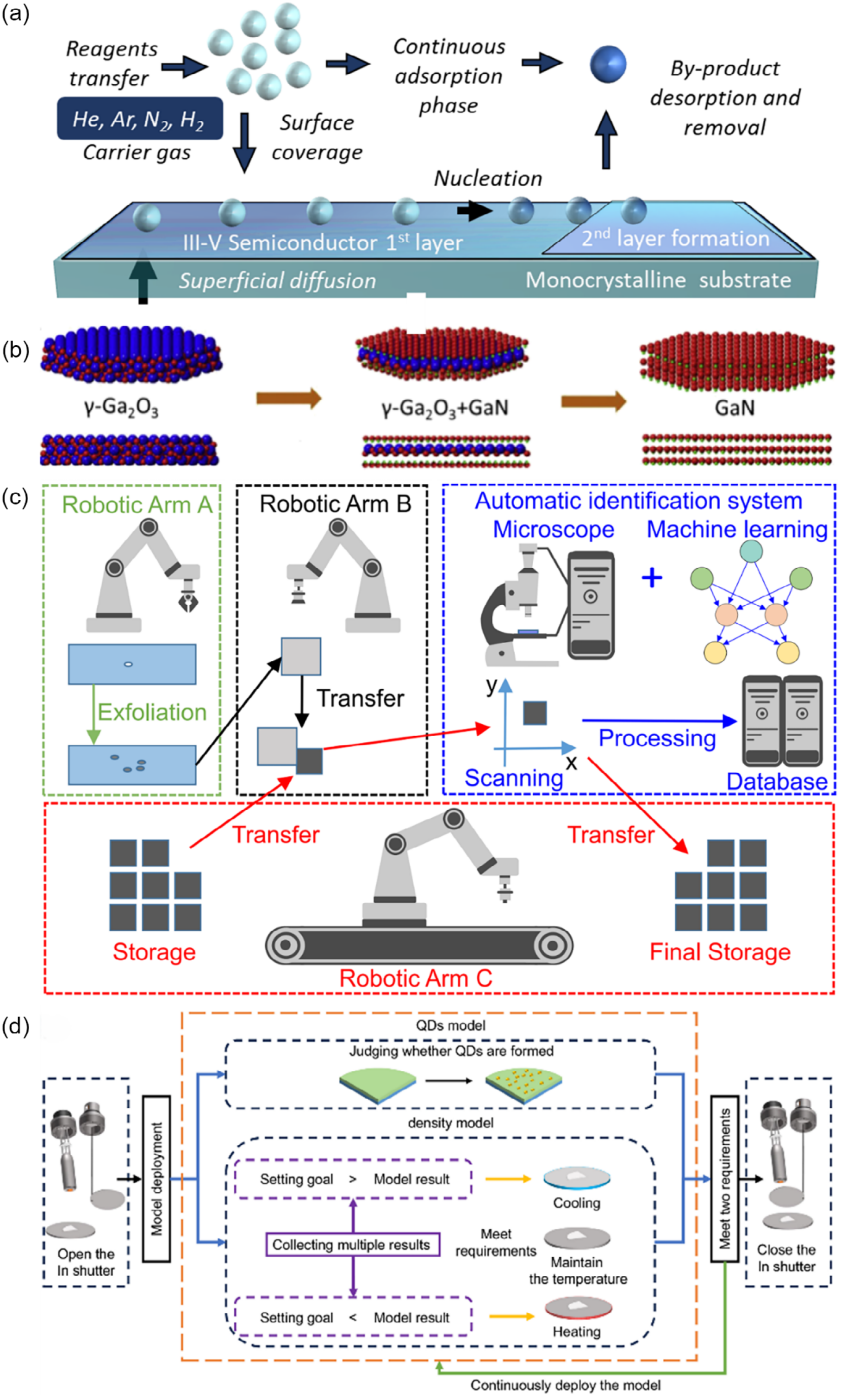
(a) The chemical vapor deposition mechanism for forming III–V layers on monocrystalline substrates. (b) Schematic of atomic substitution conversion of Ga_2_O_3_ to 2D GaN crystal (III–V semiconductor), with a magnified transmission electron microscopy (TEM) image of Ga_2_O_3_ nanosheets. Reproduced with permission from ref. [112]. Copyright 2017, American Chemical Society. (c) Schematic representation of the 2D monolayer exfoliation and searching process. Initially, the single crystals of 2D materials on the blue‐tape are repetitively folded by robotic arm‐A. Subsequently, the small flakes of 2D materials are transferred to the Si substrate by the designed stamp of the robotic arm‐B. An automatic detection system based on machine learning is employed to identify the 2D monolayers and catalog their position in a database. Subsequently, the Si substrate with monolayer 2D materials is storage. Reproduced with permission from ref. [113] Copyright 2025, American Chemical Society. (d) A control logic diagram for the model deployment. After opening the shutter, the substrate temperature remains unchanged if the label output from the density model aligns with the preset target before quantum dot (QD) formation. If the output from the density model exceeds the target density, the substrate temperature increases; otherwise, it decreases. Once the QDs model recognizes QD formation, the shuttle will remain open until the density model's output matches the target. At this point, it closes the shutter to complete the growth process. Reproduced from ref. [114] under the terms of the CC BY license.

Liquid phase epitaxy (LPE), another extension of the epitaxy technique, deposits monocrystalline layers from a highly saturated liquid solution by direct precipitation. Owing to their suitable melting temperatures, LPE is used for antimony, gallium, and indium‐based III–V compounds [54, 119, 120, 121, 122]. While LPE offers simplicity and control over product quality, it cannot use high‐melting III–V materials as solvents, and layer growth rate control is challenging. Molecular beam epitaxy (MBE) is ideal for producing ultra‐thin III–V semiconductor layers through the co‐evaporation of constituent elements in an ultra‐high vacuum (10^–10^ torr), allowing precise control over crystalline quality, layer uniformity, chemical composition, and doping. However, MBE is slow and expensive [122, 123]. Pan et al. used MBE to grow single‐crystalline InSb nanosheets on Si (111) substrate via MBE [124]. In this work, Ag was deposited on the Si (111) substrate as a catalyst, and the beam equivalent pressure ratio of As/In was adjusted to grow InAs nanowires. Subsequently, the Sb/In source was switched to grow InSb over the InAs nanowire stems. As growth time increased, the size of the InSb nanosheets expanded both vertically and laterally.

Recently, the atomic substitution technique has been effectively used to develop 2D III–V semiconductors [125]. The choice of parent material is crucial for maintaining the optoelectrical characteristics of the final product. Liu et al. synthesized mesoporous 2D GaN using this method [112]. A 5–10 nm γ‐Ga_2_O_3_ layer (metastable) with a smooth surface, high crystallinity, and uniform thickness was prepared via a hydrothermal method as a precursor. This γ‐Ga_2_O_3_ layer was then nitridated with NH_3_, converting the oxygen into the hexagonal GaN (Figure 4b). γ‐Ga_2_O_3_ plays a key role in the structural transformation due to its unstable structure and lattice compatibility with both the (220) crystal plane of the cubic structure and the (100) plane of hexagonal GaN. Similarly, GaS and GaSe can also be nitridated to form their respective III–V semiconductors.

ML‐assisted growth of III–V semiconductors could pave the way to achieve highly crystalline and compositionally precise electrode materials for PEC‐WS. AI is emerging as a transformative tool, enabling intelligent control of the exfoliation and transfer process through real‐time parameter optimization, crack prevention, and path planning. Deep learning architectures facilitate layer identification and defect detection, while reinforcement learning enables high‐precision autonomous robotic manipulation [97]. Figure 4c illustrates schematics of how generative AI and machine learning can be employed to develop a robotic fabrication and search system for high‐speed, reproducible mechanical exfoliation and detection of monolayer 2D III–V semiconductors [113]. The AI‐driven platform comprises robotic mechanical exfoliation, automated transfer of the exfoliated flakes to a detection stage, and automatic search and identification of monolayer 2D semiconductors using an optical microscope.

The pioneering work of Shen et al. established a state‐of‐the‐art AI‐assisted MBE procedure and reported a real‐time feedback control method to realize the precise fabrication of III–V semiconductors with arbitrary density [114, 126, 127]. A ResNet‐50 ML model was trained using reflection high‐energy electron diffraction (RHEED) videos as input data. The information was assisted by real‐time feedback on surface morphologies for process control. The model successfully expedited the optimization process and reproducibility of III–V semiconductors with exact compositions. The deployment of the ResNet50 model for III–V QDs, is schematically described in Figure 4d. These early‐stage case studies underscore the wide potential of employing ML and generative AI tools to develop high‐quality semiconductor materials of any type.

The use of AI and ML in utilizing III–V semiconductors has immense potential. However, the first major shortage is the lack of large, standardized, high‐quality datasets. The techniques needed to generate high‐quality data, namely epitaxial growth techniques such as MBE and MOCVD, are so expensive that high‐throughput data generation is financially prohibitive. Additionally, many deep learning algorithms are “black‐box” in nature, which makes it difficult to identify the basic thermodynamic and kinetic mechanisms responsible for crystal growth, thereby hindering rational materials design. The future development of physics‐informed neural networks (PINNs), which codify established crystallographic rules into the learning, will be crucial. In conclusion, the field is moving toward self‐driving laboratories in which the AI controls synthesis parameters, performs in situ characterization, and optimizes growth recipes in a real‐time, iterative manner. This will accelerate the rate of discovery of scalable III–V photoelectrodes for PEC‐WS.

## 4 Mechanism‐Based Photocorrosion Inhibition

As previously discussed, III–V semiconductor‐based devices show promising photocurrent densities during PEC‐WS. However, their practical application is significantly limited by inherent thermodynamic instability and susceptibility to photocorrosion, and high production cost. The interfacial properties between the photoelectrode and the electrolyte are crucial for enhancing charge carrier utilization. Effective PEC‐WS requires electrolyte molecules to interact closely with the electrode surface, making surface wettability (hydrophilicity/hydrophobicity) a key performance factor. Tunable surface wettability of III–V photoelectrodes facilitates efficient gas bubble release, thereby significantly enhancing overall water‐splitting efficiency. Optimizing wettability also facilitates water molecule adsorption, thereby enhancing H_2_ generation [82]. Excessive hydrophobicity at the electrode interface can impede light penetration and hinder electrolyte access to porous structures, thereby reducing photocurrent.

Recently, Domen et al. demonstrated that electrode hydrophilicity affects light penetration and gas bubble size during evolution [128]. Bubble size significantly affects water‐molecule adhesion to the electrode surface, thereby impacting overall electrode performance. The Young–Duprè equation, which models the relationship between the contact angle, surface tension, and adhesion forces of a bubble or droplet on an electrode surface (wettability), shows that larger contact angles reduce adhesion and enhance bubble detachment, and vice versa, on flat surfaces [129]. These dynamics suggest that, alongside optoelectronic characteristics, the surface wettability can be tuned through various modifications to achieve durable PEC‐WS.

Various modification strategies have been developed to inhibit photocorrosion in Group III–V semiconductor electrodes. These include (i) controlling the phase and morphology of photoelectrode materials, (ii) applying surface protection to enhance the photostability of PEC devices (especially under high pH and anodic conditions), and (iii) modifying parent III–V semiconductors with complementary materials. Also, understanding electrode/electrolyte interactions and the effects of physicochemical parameters on photostability is crucial. The following sections outline key strategies for achieving stable, durable photoelectrodes. We also provide extensive machine learning context to enhance the practical implications and predictive design of each strategy.

### 4.1 Crystal and Facet Control Growth of III–V Materials

The intrinsic facet‐controlled growth and crystallinity dictate the final extrinsic morphology of III–V semiconductors and their overall optoelectronic characteristics. Different planes of atoms are arranged in specific directions to create 0D, 1D, or higher‐dimensional macroscopic shapes (morphologies) [33, 53, 130]. 1D materials such as nanowires, nanorods, nanotubes, and nanopencils, etc., are widely regarded as ideal for photo‐driven applications owing to their selectively exposed facets and superior optoelectronic properties [131, 132, 133]. Nanowire‐shaped III–V semiconductors, such as GaAs nanowires (NWs), GaInP_2_, GaInP_2_ NWs, and GaSb NWs [51, 105, 134], etc., exhibit excellent PEC‐WS performance due to enhanced visible‐light absorption, large surface areas, effective light trapping, and efficient carrier extraction [100, 134, 135].

Moreover, the orientation of exposed crystal facets in crystals significantly affects photostability and overall device performance. For example, exposing the (010) facet enhances the rate‐limiting PEC water oxidation reaction by promoting hole transport, as shown by the model photocatalyst “monoclinic scheelite bismuth vanadate (m‐BiVO_4_)” (Figure 5a–d) [29, 139]. Consequently, crystal facet and phase engineering have been adopted to regulate the morphology and intrinsic properties of III–V semiconductors and other photoactive materials [140].

**FIGURE 5:**
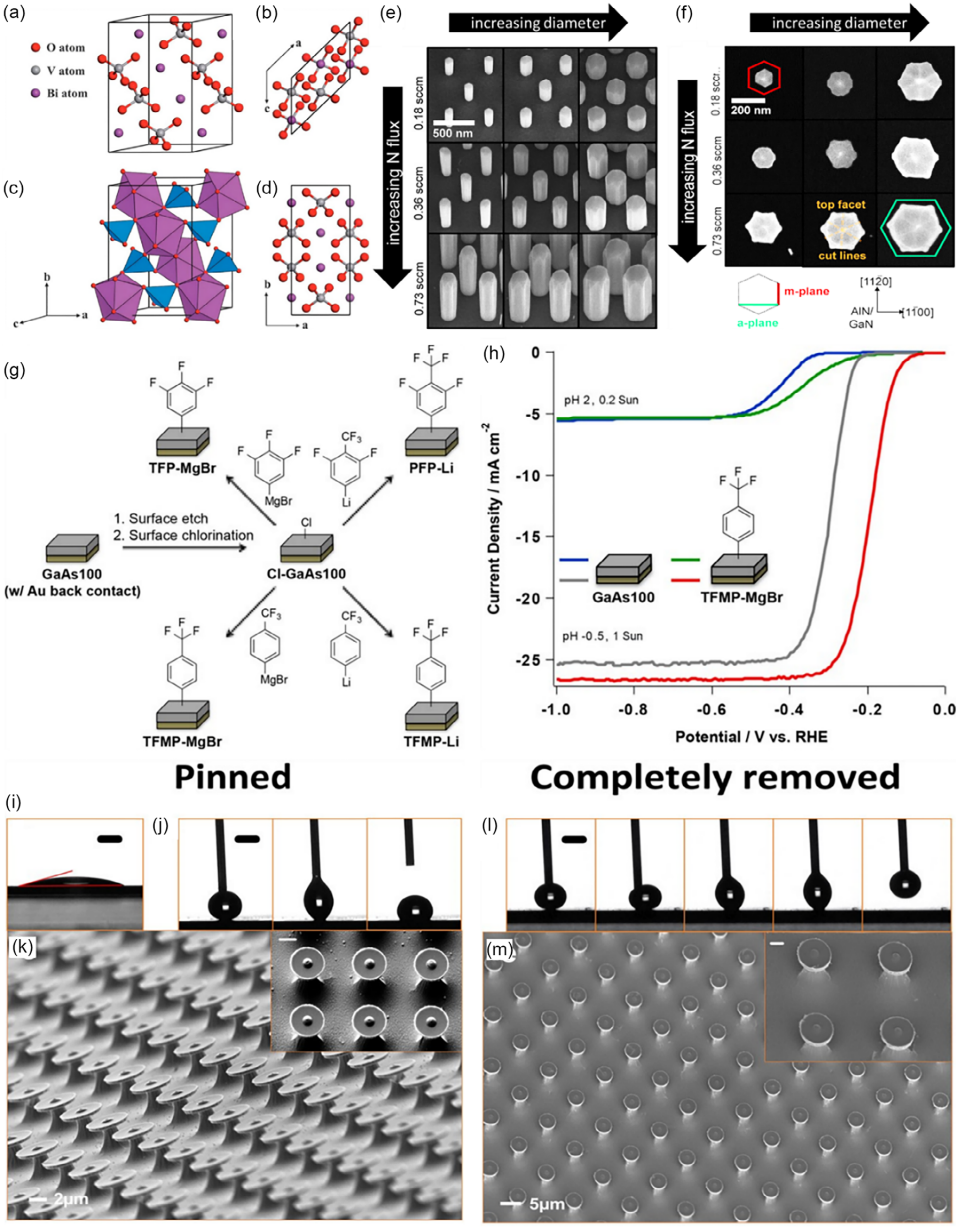
(a–d) Facet‐controlled mBiVO_4_: (a) mBiVO_4_ crystal, (b) polyhedron structure of mBiVO_4_ (purple dodecahedron = BiO_8_, blue tetrahedron = VO_4_), (c) top view, and (d) side view of mBiVO_4_ crystal. Reproduced with permission from ref. [136]. Copyright 2011 Royal Society of Chemistry. (e) Tilted 45° SEM images of GaN NWs: Size variation with N fluxes of 0.18, 0.36, and 0.73 SCCM. (f) Top‐view SEM images of GaN NWs: N flux also affects the diameter. The circled hexagons indicate the wurtzite m‐(a‐) plane orientations. Reproduced with permission from ref. [53]. Copyright 2022, AIP Publishing. (g) Schematic of (100) exposed p‐GaAs modification via surface dipoles. (h) Linear sweep voltammetry (LSV) curves of bare GaAs100 and TFMP‐MgBr photocathodes analyzed under pH 2/0.2 Sun (blue/green plots) and pH 0.5/1 Sun (dark gray/red plots) illumination. Reproduced with permission from ref. [137]. Copyright 2017, Wiley‐VCH. (i–m) InP/InGaAs/InP surface morphologies and wettability: (i) Water drop on flat InP wafer (highly hydrophilic with contact angle <23°, scale bar = 1 mm). Sample 1 (deep re‐entrant holes): (j) Water drop on patterned InP surface (*H* = 2.5 μm, *D* = 4.5 μm, *L* = 8 μm, *λ*_1_ = 0.249, contact angle = 124.5°). (k) SEM images of the patterned surface of sample 1 with a top view (inset, scale bar = 2 µm). Sample 2 (shallow re‐entrant holes): (l) Water drop on patterned InP surface (*H* = 2 μm, *D* = 4 μm, *L* = 10 μm, *λ*_2_ = 0.126, scale bar = 1 mm). (m) SEM images of the patterned surface of sample 2 with a top view (inset, scale bar = 2 µm). Reproduced from ref. [138] under the terms of the CC BY license. N, nitrogen; SEM, scanning electron microscopy.

Preliminary studies on exposed facets were based on the Gibbs–Wulff theorem, which explains how selective nucleation and preferential facet growth minimize total surface energy. According to this principle, facets with higher surface energy grow faster and eventually disappear as the final crystals form, whereas lower‐energy facets grow more slowly and remain in the final crystal structure [141, 142, 143]. This concept was experimentally applied to III–V semiconductors, which commonly exhibit zinc blende and wurtzite crystal structures. The morphology of GaN nanowires (NWs) was tuned by adjusting the III–V ratio during selective area growth via MBE. As shown in Figure 5e,f, nitrogen flux regulated crystal facet growth kinetics along the NW sidewalls. However, the resulting crystals exhibited several defects and faults due to internal stress, which impaired their optoelectronic performance [53]. Therefore, improper control of 1D morphology can introduce charge trapping centers and interfacial energy barriers, leading to photocorrosion and reduced STH efficiency. To mitigate stress and associated defects, a textured GaP template was monolithically integrated onto a Si surface, enhancing PEC‐WS performance. The stability of the (114)A facets was first verified using scanning tunneling microscopy, TEM, and atomic force microscopy. Density functional theory (DFT) calculations then confirmed that MBE produced a stress‐free nanopatterned surface with regular (114)A GaP facets on the Si substrate. This (114)A GaP/Si configuration offers benefits such as increased surface area, improved ohmic contact, and favorable band alignment for improved PEC‐WS [144].

Exposed crystal facets of III–V materials are readily modifiable with other materials. Garner et al. observed that p‐GaAs(100) surfaces can be covalently fluorinated with aromatic molecules to effectively tune interfacial energetics in highly acidic electrolytes. The (100)‐oriented p‐GaAs substrates were etched with concentrated H_2_SO_4_ for 30 s, rinsed with water, and dried under a N_2_ stream (Figure 5g). This surface was then chlorinated with 2 M HCl in diethyl ether under an N_2_ atmosphere for 50 min, and the resulting surface was used immediately. Owing to their high surface energy, p‐GaAs(100) facets readily underwent fluorination by chlorine substitution**.** The facet‐specific modification significantly improved PEC performance, shifting the PEC‐HER onset potential anodically and enhancing photostability by delaying arsenic oxide formation (Figure 5h) [137].

Photoelectrode wettability also largely depends on interfacial morphology, which is shaped by the crystal growth sequence. For example, GaAs morphology during hydride vapor phase epitaxy was influenced by growth temperature and substrate orientation, indicating that nonfaceted growth occurred at higher temperatures, while faceted GaAs crystals formed at lower temperatures [145]. Siabi et al. experimentally investigated the correlation between surface roughness and contact angle by sputtering silver‐based thin films onto substrates with controlled inclination angles using shadowing objects [146]. Shadowing decreased the contact angle of thin films, while high surface roughness and porosity enhanced it. Similarly, patterning the interfacial morphology of III–V semiconductors can optimize wettability. For instance, an InP/InGaAs/InP multilayer array was patterned via electron‐beam lithography to adjust its wettability, which was predicted by modeling adhesion energies between the water droplet, needle, and patterned surface. Water‐repellent behavior is essential for stable optical applications. Surface patterning and roughness determine the wettability of different samples (Figure 5i–m). A droplet (approximately 2 μL) remained pinned on the deep‐holed InP/InGaAs/InP surface (Sample 1, Figure 5i–k) but made weak contact with the shallow‐holed re‐entrant surface (Sample 2, Figure 5l,m), as confirmed by the solid surface/drop contact fraction values. Here, *λ*_1_ and *λ*_2_ represent the contact fractions for Samples 1 and 2, respectively, with *λ*_1_ > *λ*_2,_ indicating easier droplet removal from Sample 2 due to minimal pinning [138].

The static contact angle measurements can provide a useful preliminary representation for the surface hydrophilicity but must be interpreted carefully. A small contact angle of the droplet should not be equated directly with gas‐bubble detachment during actual PEC operation. Static measurements are limited by the partial neglect of the operando effects on the total capillary force contribution from parameters such as contact‐angle hysteresis, three‐phase contact‐line pinning, dynamic surface‐roughness effects, and the fluid dynamics of continuous gas evolution. Gas bubbles that remain attached to the surface due to high hysteresis hinder the active catalytic sites and increase local mass‐transport resistance. It also creates a local point of stress, which can speed up the photocorrosion process. Engineering a hydrophilic surface is beneficial; however, to truly mitigate photocorrosion via bubble management, one must evaluate the dynamic bubble release radius and residence time during active water‐splitting conditions.

Controlling electrode wettability may enhance the PEC performance of faceted III–V semiconductors, warranting further large‐scale studies. Table 2 summarizes the optoelectrical and photoactivity performance of faceted III–V materials. A comparison of PEC‐WS results for bulk, planar, and faceted photoelectrodes shows that facet exposure greatly influences device performance. Nonetheless, achieving precise crystal growth with specific facets is a huge challenge in synthetic chemistry.

**TABLE 2:** Crystal and facet‐directed growth and their impacts on the overall photoelectrochemical and related optoelectrical properties of III–V semiconductors.

Photoelectrode composition	Exposed facet	Electrolyte (Conc., pH)	Water‐splitting and efficiency under 1‐SUN		References
MOCVD integrated bifacial GaAs wafers	(100)	H_2_SO_4_ 0.5 M, pH = 0.55	*I*_sc_ = 21.8 mAcm^−2^ @ −0.5 V_RHE_ *V*_onset_ = 0.936 V_RHE_ *η*_STH_ = 2.5% ff = 13.4%		[147]
HVPE grew GaAs substrates	(100) and (110)	Not given	GaAs (110) *I*_sc_ = 16.2 mAcm^−2^ *V*_oc_ = 0.996 V ff = 79.7%	GaAs (100) *I*_sc_ = 17.2 mAcm^−2^ *V*_oc_ = 0.996 V ff = 81.5%	[148]
Covalent surface‐modified GaAs	(100)	H_2_SO_4_ 3.0 M, pH = 0.5	Pristine GaAs (100) *I*_sc_ = 24.8 mAcm^−2^ *V*_onset_ = 0.26 V_RHE_ Photostability = ~30 h	Halogenated GaAs (100) *I*_sc_ = 26.2 mAcm^−2^ *V*_onset_ = 0.23 V_RHE_ Photostability = ~50 h	[137]
Wurtzite GaP NWs grew by soft nanoimprint lithography	(111)	HClO_4_ 1.0 M, pH = 0.5	Planar GaP *I*_sc_ = 1.18 mAcm^−2^ *V*_oc_ = 0.68 V Energy conversion‐ efficiency = 0.17% ff = 21%	GaP (111) NWs *I*_sc_ = 6.41 mAcm^−2^ *V*_oc_ = 0.71 V Energy conversion‐ efficiency = 1.50% ff = 33%	[149]
Surfactant‐free, self‐seeded GaP NWs	(111)	Acetate buffer solution 0.1 M, pH = 5.2	H_2_ production rate = ∼9 nmol h^−1^ Time = 18 h, Exhibited ∼0.15 V more positive potential under light		[150]
Polar‐surface‐dominated zigzag GaN NWs	(1011) N‐terminated Facet	Na_2_SO_4_ 0.1 M, pH = 7.0 in aq. CH_3_OH	Smooth GaN *I*_sc_ = 0.04 mAcm^−2^ *V*_onset_ = 0.3 V_SCE_ H_2_ production = 3.4 μmol h^− 1^	GaN (1001) zigzag NWs *I*_sc_ = 0.1 mAcm^−2^ *V*_onset_ = 0.28 V_SCE_ H_2_ production = 29.4 μmol h^− 1^	[151]
MOVPE‐grown P‐rich p‐InP(100) homoepitaxial layer with p(2 × 2)/c(4 × 2) surface reconstruction	(100)	No electrolyte (tr‐2PPE in ultra‐high vacuum)	Bulk InP (literature) Bandgap = 1.34–1.35 eV HSE‐DFT bandgap = 1.35 eV	P‐rich p‐InP(100) Bandgap = 1.32 ± 0.08 eV (tr‐2PPE) Nine electronic states resolved between the VB edge and the vacuum level State cluster ≈1.6 eV above the CBM assigned to a bulk‐to‐surface transition Longest CB decay constant = 1550 fs (2.33 eV pump, 4.49 eV probe)	[44]
Single‐crystal In_0.25_Ga_0.75_N NWs by molecular beam epitaxy	Wurtzite (0002)	H_2_SO_4_ 0.5 M, pH = 1.0	*I*_ph_ = 2.8 mAcm^−2^ @ 0 V_Pt_ Photostability = ~300 h *η*_STH_ = 3.4%		[152]
CVD‐assisted InGaN NWs	Wurtzite (0002)	KOH 0.5 M, pH = 13.5	*V*_onset_ = 0.234 V_RHE_ Photostability = 3000 s @ 0.2 V_RHE_ ABPE = 1% @ 0.63 V_RHE_		[153]
Coaxial InGaN/GaN multiquantum well (MQW) NWs	GaN with (1101) dominating facet	HCl 1.0 M, pH = 1.0	Pristine GaN *I*_ph_ = 0.45 mAcm^−2^ @ 1.0 V_RHE_ *V*_onset_ = 0.6 *V*_RHE_ Photostability = 44% activity lost @ 3 h IPCE = 2.9% @ 350 nm	InGaN/GaN MQW NWs *I*_ph_ = 1.75 mAcm^−2^ @ 1.0 *V*_RHE_ *V*_onset_ = 0.8 *V*_RHE_ Photostability = 30% activity lost @ 3 h IPCE = 8.6% @ 350 nm	[154]
Ternary GaAsP core–shell NWs	(111) directed growth on Si	KPi 0.1 M, pH = 7 Na_2_SO_4_ 0.1 M, pH= 10	*I*_ph_ = 4.5 mAcm^−2^ @ 0 V_RHE_ *V*_onset_ = 0.54 V_RHE_ *η*_STH_ = 0.5% Photostability = 35 min @ 0.2 V_RHE_		[155]

Abbreviations: ABPE, applied‐bias photon‐to‐current efficiency; CB, conduction band; CBM, conduction band minimum; CVD, chemical vapor deposition; HSE‐DFT, density functional theory with the Heyd–Scuseria–Ernzerhof hybrid functional; HVPE, hydride vapor phase epitaxy; IPCE, incident photon‐to‐current conversion efficiency; MOCVD, metal‐organic chemical vapor deposition; MOVPE, metal‐organic vapor phase epitaxy; MQW, multiple quantum well; NWs, nanowires; tr‐2PPE, time‐resolved two‐photon photoemission spectroscopy; VB, valence band.

Currently, crystal structure prediction based on global optimization techniques can be an excellent tool for mapping the vast category of III–V semiconductors for PEC‐WS. The inclusion of ML in post‐growth characterization is fundamental to assessing crystal quality and generating ground‐truth labels for tasks such as defect prediction and quality classification. Figure 6a represents a conceptual diagram of a ML‐enhanced crystal growth process [156]. Generative AI could efficiently navigate larger regions of crystal chemical space informed by structure‐property datasets of III–V materials by using a modified model similar to that of Park et al. [157]. They introduced a model named “Chemeleon” which could generate chemical compositions and crystal structures by learning from both textual descriptions and 3D structural data. The model employs denoising diffusion for compound generation, using textual inputs aligned with structural data through cross‐modal contrastive learning. The schematic of this AI model was composed of two components. The first component (Figure 6b) is a text encoder pre‐trained via contrastive learning to align text embeddings with graph embedding vectors from equivariant graph neural networks (GNNs). The second component (Figure 6c) is a classifier‐free guidance denoising diffusion model for composition and structure generation, which is iteratively trained to predict the temporal evolution of noise by incorporating the text embeddings obtained from the pretrained text encoder. Employing this model to control the crystal growth of III–V materials can improve the PEC‐WS metrics. The single crystal III–V growth with controlled facets can be predicted at an industrial scale easily.

**FIGURE 6:**
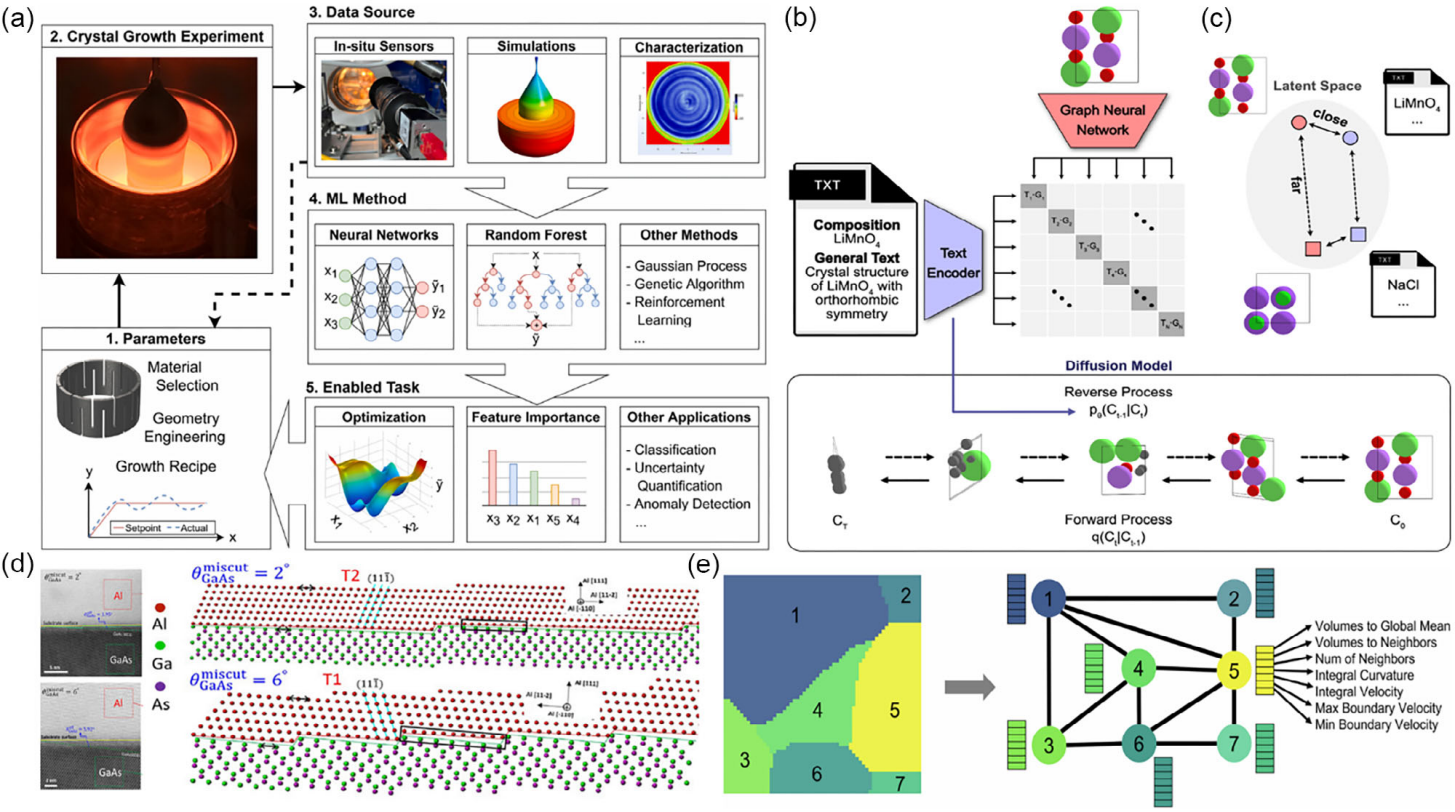
(a) Conceptual diagram of a machine‐learning‐enhanced crystal growth process. Reproduced with permission from ref. [156] Copyright 2025, Elsevier. (b) The text‐guided denoising diffusion model comprises two key components: (1) Crystal CLIP (Contrastive Language‐Image Pretraining), a text encoder pretrained through contrastive learning to align text embeddings with graph neural network (GNN) embeddings derived from crystal structures, and (2) a classifier‐free diffusion model, which iteratively predicts noise at each time step while integrating text embeddings from the pre‐trained Crystal CLIP. In this framework, *q* denotes the forward diffusion process (posterior) progressively adding noise to the crystal structures, while *p*_*θ*_ represents the reverse diffusion process (learned approximation of the posterior) aimed at generating crystal structures. *C*_t_ refers to crystal structures at time step, *t*. (c) Illustration of the contrastive learning objective in Crystal CLIP, where positive pairs, which consist of text and graph embeddings from the same crystal structures, are brought closer together in the latent space, while negative pairs are pushed further apart. Reproduced from ref. [157] under the terms of the CC BY license. (d) Atomic structures of epitaxial Al crystalline domains on miscut GaAs substrates. (Left) Scanning transmission electron microscopy (STEM) image of a 40‐nm‐thick Al film grown on a 2° and 6° miscut GaAs (001) substrate. (Right) Schematic models illustrate the atomic arrangement of Al on the surface step terrace of 2°‐miscut and 6°‐miscut GaAs (001) vicinal substrates. The misalignment between Al (111) and GaAs (001) lattice planes becomes larger at the 6° substrate miscut. The double‐headed arrows denote the spacing of the Al [111] or Ga [109] planes, while the rectangles denote the domain matching units. Reproduced with permission from ref. [158] Copyright 2026, Wiley‐VCH. (e) Visualization of a graph representation built from an N‐grain microstructure in a cropped 2D plane (N = 7). Grains are represented as nodes, and grain boundaries are edges in the graph in the right panel. The d different physical features (*d* = 7 herein) of each grain are stored in a feature vector for each node and combined in a feature matrix X. Adjacency relationship between grains are stored in an adjacency matrix A, yielding a graph representation G = (X, A). Reproduced from ref. [159] under the terms of the CC BY license.

However, in polycrystalline III–V materials, defined‐facet growth is often inhibited by abnormal grain growth caused by misaligned facet material selection and improper synthesis handling. The rapid and disproportionate enlargement of crystal growth causes an abnormal grain environment that can lead to deleterious changes in the crystal structure, leading to insufficient PEC‐WS performance. Dislocations, structures/grain boundaries, and crystal mismatch in polycrystalline III–V materials are well documented [106, 160]. For instance, epitaxial Al nanofilms on GaAs substrates exhibit serious mismatching and dislocation at varying miscut angles [158]. High‐resolution STEM analyses supported by the 3D model in Figure 6d explore the microstructure of epitaxial Al nanofilms on GaAs substrates with varying miscut angles. Geometric considerations of the lattice matching between Al [111] and Ga [109] planes with spacing 4.96 and 4.00 Å, respectively, are greatly affected by the miscut angle of the GaAs substrate. The dislocations will not be active under suitable conditions. The Al layers grown initially are taken to introduce strain to the grown layers. When the miscut angle is 2°, the Al (111) crystal is well matched to the GaAs (001) substrate. As the miscut angle of the substrate is increased to 2°, the terrace width corresponds to around 20 [11] Ga spacings (~80 Å), while at 6°, it becomes roughly 6 [109] Ga spacings (~24 Å). As presented in the upper panel of Figure 6d for the case of 2° miscut, 4/5 domain matching remains effective despite a small misalignment angle of approximately 0.4° between the Al film and GaAs substrate. In contrast, for the situation of 6° miscut (lower panel), the matching condition shifts to five [111] Al spacings against six [109] Ga spacings (5/6 domain matching) as a result of a larger Al (111)/GaAs (001) misalignment and narrower terrace widths (also highlighted by the rectangle). This results in an increased lattice mismatch of 0.80 Å and a stronger compressive strain than in the Al/2°‐miscut GaAs sample [158]. These resultant lattice strains and dislocations act as critical charge recombination centers and primary sites for anodic photocorrosion.

In this scenario, a predictive model could detect abnormal grain growth in the initial phase of fabrication, thereby adding substantial economic value to the synthesis. Variations in miscut values can severely affect the photophysical and mechanical properties of III–V substrates, rendering them highly susceptible to severe photocorrosion. Therefore, a method for predicting grain abnormality in the early stages of crystal development is highly desirable. In this regard, Zhou et al. established ML‐enhanced AI predictive methods. These methods interconnect grain–grain interactions with grain properties realized from the spatiotemporal evolution of grain characteristics (Figure 6e). The first, PAL (predicting abnormality with LSTM), analyzes grain features using a long short‐term memory (LSTM) network, and the second, PAGL (predicting abnormality with GCRN and LSTM), supplements the LSTM with a graph‐based convolutional recurrent network (GCRN). After employing these predictive models on various crystal systems, it was realized that PAL and PAGL achieve high sensitivity and precision and, critically, that they can predict future abnormality long before it occurs. These models can be extended to the development of polycrystalline III–V semiconductor materials to inhibit extrinsic as well as intrinsic photocorrosion.

Predicting miscut angles and facet changes using machine learning frameworks could significantly transform the field. However, it is still difficult to verify these in silico results experimentally. The LSTM and GCRN models can make accurate predictions, but only according to their training datasets. Typically, such datasets do not contain real thermodynamic complex species that frequently occur during any real III–V synthesis. Advanced characterization techniques such as aberration‐corrected HAADF–STEM and operando XAS are required to create high‐fidelity ground truth data on atomic‐scale mismatches and transient defect states for the training algorithms [159]. The predictions made by predictive models can be calibrated through high‐confidence spectroscopic and microscopic validation. This facilitates the transition from theory‐extrapolated to highly durable defect‐controlled PEC‐WS architectures facilitated by ML crystal growth.

### 4.2 Surface Passivation of III–V Semiconductor Photoelectrodes

Surface deterioration and catalyst mass loss are key challenges for III–V photoelectrodes during high‐bias PEC‐WS. The lower bandgaps and direct electrolytic contact of III–V materials trigger interfacial photocorrosion during long‐term operation. While extensive surface functionalization can enhance PEC‐WS performance, it often slows the process due to system incompatibilities. Thus, intrinsic and extrinsic surface passivation with a suitable method offers a better solution for protecting highly reactive III–V electrode surfaces. Surface passivation was initially adopted to prevent photocorrosion and reduce electron–hole recombination by suppressing the formation of intra‐bandgap states, thereby enhancing long‐term photostability and PEC‐WS performance under harsh conditions [161, 162].

#### 4.2.1 Intrinsic Surface Passivation

Intrinsic surface passivation involves modifying the native surface atoms or bonds of the III–V material itself, without depositing a thick foreign material layer. It is crucial to strictly distinguish between uncontrolled native oxide formation and deliberately engineered interfacial chemistry. In many III–V systems, naturally forming native oxides are nonstoichiometric, thermodynamically unstable, and electronically detrimental, as they introduce severe interface states and recombination‐active defects [163, 164]. Therefore, effective intrinsic passivation requires highly controlled chemical treatments rather than generic oxidation [165].

Gallium nitride (GaN) is often cited as an exception to the generally poor performance of intrinsic oxides, owing to its wide bandgap and superior chemical stability. Research on GaN electrodes in an NaCl electrolyte demonstrated that the as‐grown surface oxide (confirmed by X‐ray photoelectron spectroscopy (XPS) analysis) could passivate the surface against corrosion and lead to photostability >4 h [166]. However, this benefit is electrolyte‐specific (near‐neutral NaCl) and does not generalize to the harsher acidic or strongly alkaline conditions required for overall water splitting. Under harsh conditions, the native oxide layer dissolves, exposing the GaN interface, which is highly susceptible to photocorrosion. Critically, Chen et al. later showed that the naturally oxidized gallium oxide on GaN nanowires acts as a carrier trapping center, preventing the transfer of photogenerated holes; PEC performance was significantly enhanced by KOH treatment that removed this native oxide [167]. Thus, even for GaN, the native oxide is not universally beneficial and often acts as a recombination center.

InP native oxides are known to cause less electrical and optical losses than other III–V oxides, making them a subject of significant research. Photoanodic oxidation of InP in H_2_SO_4_ and HCl solution has shown that passivation is favored by higher light intensity and lower proton and Cl^−^ ion concentrations, yielding a passive bilayer consisting mainly of InPO_4_ and In(PO_3_)_3_ glass‐like components [168]. This contrasts sharply with GaAs, which forms a strongly nonstoichiometric porous hydroxide. Similarly, May et al. [169] demonstrated efficient direct 14% STH conversion (reach to 17% in potentiostatic conditions) through controlled in situ interface transformation and surface functionalization of an AlInP‐containing III–V tandem structure, involving oxide/phosphate/phosphite‐type surface species and chemical/electronic passivation. Nevertheless, while InP represents a rare case where an intrinsic oxide can be engineered to provide some benefit, the process is highly condition‐dependent (requiring tight control over light intensity, pH, and ion concentration) and is difficult to control reliably.

Similarly, controlled thermal oxidation can be employed to form a thin, stoichiometric native oxide (although achieving high quality remains challenging for III–V materials). This intrinsic modification leads to significant inhibition of photocorrosion by improving the surface recombination velocity from ~10^6^ cm/s to ~10^4^–10^5^ cm/s. This was further corroborated by the thermal oxidation of GaN at 950 °C in various oxygen environments. Three types of oxygen environments, including dry oxygen, wet oxygen, and water vapor, were selected for thermal oxidation for up to 20 min to introduce native oxide layers [170]. Different surface morphologies were observed for different oxygen environments. Smooth and featureless for vapor oxidation, and grainy for dry and wet modes. Similarly, the growth rate was slowest in the vapor mode and fastest in the dry mode. Optical studies of the structures electronic properties revealed that only the vapor process did not degrade the material underneath the oxide layer, which is supported by the secondary ion mass spectroscopy (SIMS) studies, and energy‐dispersive X‐ray spectroscopy (EDS) analysis (Figure 7a–d). Dry and wet oxidation modes introduce a high level of oxygen (∼10^7^ cts/s) up to the GaN/GaN:Si layer interface. Comparatively, vapor oxidation, with its thinnest oxide layer, introduces a significantly lower amount of oxygen, with the SIMS signal level dropping from 10^6^ cts/s to below 10^5^ cts/s (close to the GaN/GaN:Si layers interface). The N signal in vapor oxidation remained unchanged, which indicated insignificant O displacement by N atoms. In the context of PEC water splitting, vaporous oxidation yields superior performance by suppressing charge recombination while preserving the interfacial chemical integrity of GaN.

**FIGURE 7:**
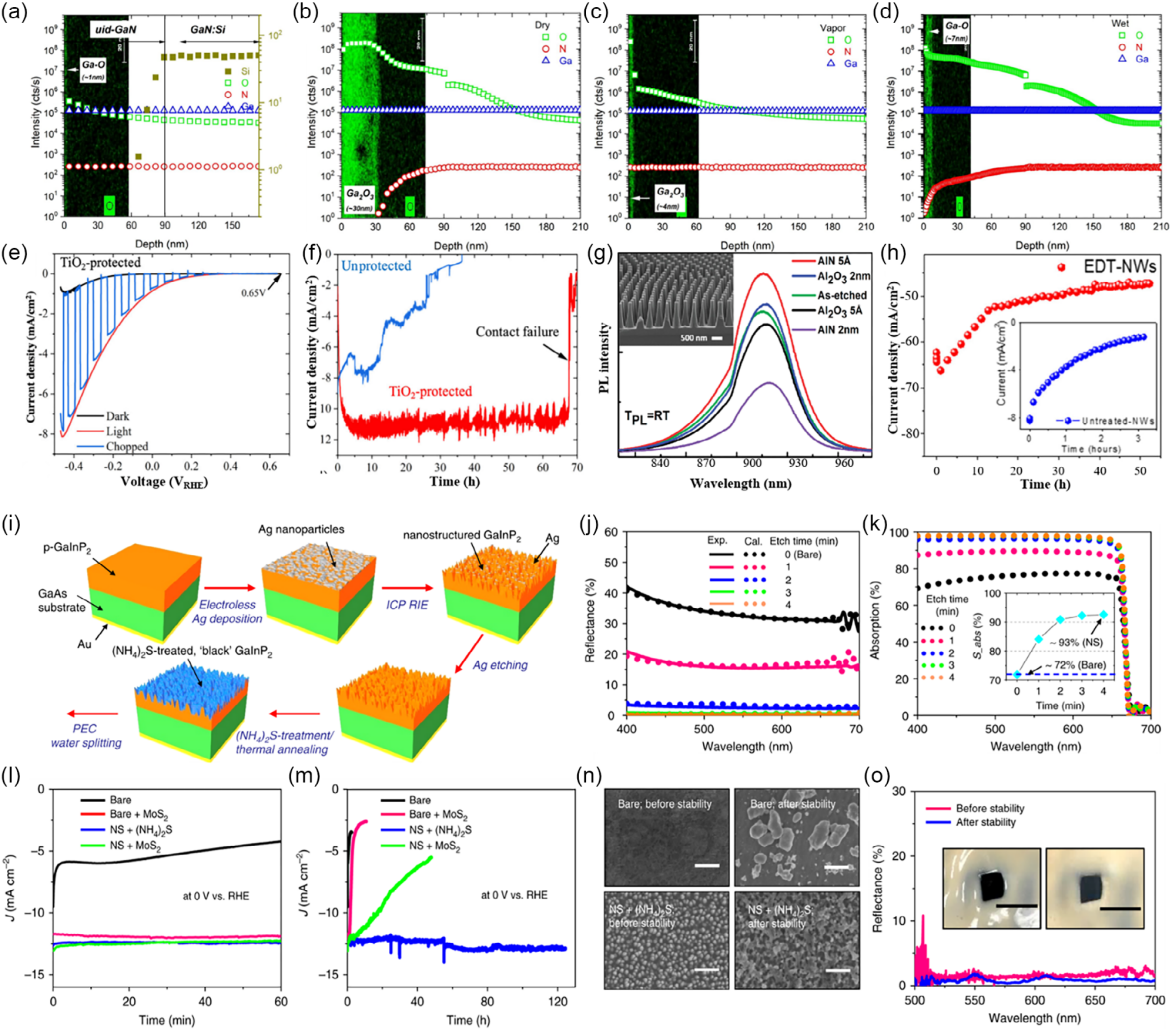
(a–d) Secondary ion mass spectrometry (SIMS) depth profiles of (a) the reference GaN sample and of samples oxidized for 20 min in (b) dry, (c) vapor, and (d) wet processes. The energy‐dispersive X‐ray spectroscopy (EDS) maps in the background were used for oxide thickness calibration. Reproduced with permission from ref. [170] Copyright 2022, Elsevier. (e,f) PEC‐WS properties of TiO_2_‐passivated and non‐passivated GaAs NW photocathodes in 0.5 M H_2_SO_4_ (pH 1) under 1 sun illumination (AM 1.5G, 100 mW cm^−2^). (e) Current density versus potential curves of the TiO_2_‐passivated photocathode in the dark, under continuous illumination, and under chopped illumination. (f) Photostability of the passivated and non‐passivated photocathodes at −0.6 V_RHE_. Reproduced with permission from ref. [134]. Copyright 2021, American Chemical Society. (g) Room‐temperature PL spectra of as‐etched InP nanopillars and of nanopillars capped with plasma‐enhanced ALD (PEALD) AlN (5 Å and 2 nm) and thermal ALD Al_2_O_3_ (5 Å and 2 nm). The PL maxima at 920–925 nm correspond to the InP band‐edge emission recorded with an InGaAs detector. The inset shows an SEM image of the as‐etched InP nanopillars (scale bar 500 nm). Reproduced from ref. [171] under the terms of the CC BY license. (h) Photocurrent versus time for 1,2‐ethanedithiol (EDT)‐treated GaN NWs at −0.4 V and pH 0. The inset shows the untreated GaN NWs measured under the same conditions. Reproduced with permission from ref. [172]. Copyright 2017, American Chemical Society. (i–o) Surface‐tailored black GaInP_2_ photocathodes. (i) Fabrication procedure comprising electroless Ag deposition, inductively coupled plasma reactive ion etching (ICP RIE), Ag etching, and (NH_4_)_2_S treatment with thermal annealing. (j) Measured (solid lines) and finite‐difference time‐domain (FDTD)‐calculated (dotted lines) total reflectance spectra of nanostructured GaInP_2_ after 0 (bare), 1, 2, 3, and 4 min of etching, recorded with an integrating sphere at an incidence angle of 8°. (k) Calculated absorption spectra of the same samples in water obtained with the numerical model validated in (j). The inset shows the integrated solar flux absorption (S_abs_) as a function of etch time. (l,m) Current density versus time plots at 0 V_RHE_ in 0.5 M H_2_SO_4_ under simulated AM 1.5G illumination for (l) short‐term and (m) long‐term measurements of bare GaInP_2_ (black), bare GaInP_2_ with MoS_2_ (red), nanostructured GaInP_2_ with (NH_4_)_2_S treatment (blue), and nanostructured GaInP_2_ with MoS_2_ but without (NH_4_)_2_S treatment (green). Dry etching and (NH_4_)_2_S treatment were performed for 4 and 15 min, respectively. (n) Top‐view SEM images of bare and nanostructured (NH_4_)_2_S‐treated GaInP_2_ before and after the stability test in (m), that is, about 1 h for the bare sample and about 124 h for the nanostructured sample (scale bars 500 nm). (o) Reflectance spectra of the nanostructured (NH_4_)_2_S‐treated photocathode before and after the stability test in (m). The insets show photographs of the samples (scale bars 5 mm). Reproduced from ref. [51] under the terms of the CC BY license.

#### 4.2.2 Extrinsic Surface Passivation

Alternatively, surface photocorrosion can be mitigated by extrinsic surface passivation. Extrinsic surface passivation is a technique used to improve the performance of semiconductor materials in optoelectrical devices. Depending on the quality and age of the III–V semiconductors, they may have defects or imperfections that serve as sites where charge carriers, such as electrons and holes, are captured and recombine, thereby accelerating photocorrosion [173, 174, 175]. Generally, the high‐quality epitaxial III–V surfaces could have limited (or no) pre‐existing/structural defects. Importantly, an atomically perfect III–V surface is thermodynamically unstable in contact with an aqueous electrolyte under illumination and applied bias, leading to photo‐induced dissolution or oxidation due to intrinsic thermodynamic and chemical instability. The corrosion process itself is not passive and creates surface roughening, pits, and new defect states, which can accelerate further degradation. This highlights the dynamic nature of the stability problem. Thus, surface engineering (protection/passivation) is the key for prolonged PEC‐WS operations.

Extrinsic surface passivation consists of the deposition of another material (metal oxide, polymer, coating, etc.) onto the semiconductor surface, at a controlled thickness, to form a discrete external layer. This layer stops the trapping of charge carriers and distinctively neutralizes surface defects on the semiconductor. It will also prevent dangerous chemical reactions, which can be harmful to the surface of the semiconductor. In contrast to intrinsic surface passivation, extrinsic passivation involves externally introduced charges or species that interact with the surface primarily through field effects, though physical interactions may still occur. The key distinction lies not in the presence or absence of physical interaction, but in whether the passivation originates from the intrinsic properties of the dielectric layer or is induced by an external source. This is achieved in two major ways: chemical passivation, where a highly transparent thin coating of a suitable material is applied, or surface chemistry alterations using chemical treatments, and physical passivation, where defects are reduced through physical methods such as strain relaxation, laser treatment, temperature, or adhesive tape. Additionally, field‐effect and energetic passivation are newer methods developed to prevent photocorrosion in optical devices [176].

The surface coating strategy, established in the late 1970s, remains the most effective method to enhance photoelectrode durability [46, 176]. Researchers have investigated various active coating materials, including semiconductor and metal oxides (such as TiO_2_, Al_2_O_3_, V_2_O_5_, ZnO, SiO_2_, NiO_*x*_, and MoO_*x*_) [46, 171], chalcogenides (mostly S and Se) [177], group III–V materials (such as GaN) [178], and organic solutions [172, 179]. A 7 nm TiO_2_ thin layer, applied via atomic layer deposition (ALD) to III–V NW electrodes, inhibits photocorrosion and notably improves photostability (Figure 7e,f). TiO_2_‐passivated GaAs NWs maintain 91.4% photoluminescence intensity after 14 months under ambient conditions. This photostability resulted from the robust, pinhole‐free deposition of the TiO_2_ layer. TiO_2_‐passivated GaAs NWs, utilized as photocathodes for PEC‐WS, generated 45% more photocurrent density than unprotected counterparts and exhibited 91% Faraday efficiency with 67 h of PEC photostability under extreme pH (pH = 1). In contrast, non‐passivated GaAs NWs photocathodes failed after 35 h [134].

ALD is an ideal technique for fabricating thin passivation layers. Various surface‐passivating coatings, including AlN, TiN, Al_2_O_3_, GaN, and TiO_2_, were applied to III–V NWs (GaAs and InP) and InP nanopillars using low‐temperature ALD [180]. A 2 Å thick AlN passivation layer improved GaAs NWs, as shown by low‐temperature photoluminescence (PL) at 15 K, while a 2 nm thick Al_2_O_3_ layer was effective for InP NWs. For InP nanopillars, a 5 Å AlN layer and a 2 nm Al_2_O_3_ layer both enhanced the room‐temperature PL intensity relative to the as‐etched pillars, whereas a 2 nm AlN layer suppressed it (Figure 7g). The remaining ALD capping layers negatively impacted the InP surface. Optimized passivation nearly doubled the carrier decay time in InP nanopillars (from 251 to ~525 ps), and the capped nanostructures retained their optical properties after 6 months of aging [171]. GaInP_2_, a valuable III–V semiconductor with a large bandgap, is used as the top junction in dual‐absorber PEC‐WS tandem cells. However, its poor intrinsic catalytic ability and surface photocorrosion hinder its performance. A nanoscale MoS_2_ chalcogenide film efficiently protected and served as an HER catalyst for the GaInP_2_ multijunction photocathode in the PEC‐WS cell. After surface passivation, only a 10% loss in photocurrent occurred after 100 h of illumination. The enhancement is attributed to better electronic transfer and minimal photocarrier recombination [180]. Chemical surface treatment with organic compounds has also significantly enhanced efficiency. For example, 1D GaN NWs were treated with 1,2‐ethanedithiol (EDT), boosting the incident photon‐to‐current conversion efficiency (IPCE) from 8.1% to 18.3%. Surface passivation also improved photostability, as GaN NWs retained 80% of their initial photocurrent after 55 h under highly acidic conditions (Figure 7h) [172].

Treating GaAs with (NH_4_)_2_S forms a thin Ga‐S or As‐S layer that ties up dangling bonds [164]. Lim et al. exploited corrosion‐resistant surface stoichiometry and a structurally tailored reactive interface to produce nanoporous, density‐graded GaInP_2_‐(NH_4_)_2_S, which was employed at the surface for intrinsic passivation [51]. The synthesis scheme of the density‐graded GaInP_2_‐(NH_4_)_2_S nanoarchitectures is provided in Figure 7i. The reflectance and PEC‐WS photostability results in Figure 7j–m confirm the impact of intrinsic surface passivation that effectively reduced reflection and surface recombination of photogenerated carriers, leading to excellent electrochemical durability of >124 h. The post‐analysis SEM images indicated a clear deterioration in the nonpassivated base surface, which corresponds to a reflectance shift (Figure 7n,o).

#### 4.2.3 Structural, Strain, and Field‐Effect Engineering

Physical extrinsic passivation addresses defects and pinholes through strain relaxation or physical treatments, such as mechanical force, thermal annealing, adhesive tape, surface polishing, or lasers [163, 173, 177, 181]. Unlike chemical passivation, physical passivation is adaptable to various compositions and configurations, enhancing robustness against detrimental interfacial chemical reactions during PEC processes. This method has been successfully used in perovskite solar cells and shows promise for PEC‐WS applications. Thermal annealing, in particular, improves crystallinity and reduces defect sites at the III–V semiconductor film interface over time [182]. Chemical and mechanical polishing are widely employed to control the interfacial properties of III–V and Si wafer electrodes [183, 184]. Similarly, laser surface polishing, a simple yet effective physical passivation method, enhances the robustness of photoelectrode surfaces. Kong et al. demonstrated that high‐energy femtosecond laser polishing improves the spin‐coated perovskite film surface [185]. As shown in Figure 8a, the spin‐coated perovskite thin film was annealed, exposed to the high‐energy femtosecond laser, and then spin‐coated again. The laser treatment increased grain size and reduced grain boundaries, boosting device efficiency by nearly 9% compared with nonpolished surfaces due to fewer defects and lower nonradiative recombination. A systematic study on the optical and morphological characteristics of large‐area GaAs micro/nanostructures formed by femtosecond laser irradiation demonstrated that increased exposure enlarged the microstructures and degraded the overall optical performance of the III–V device [187]. Recently, Reeves et al. applied this method to III–V photovoltaic (PV) solar cells, detaching rigid, lattice‐matched GaAs devices (16 mm^2^ × 3.5 µm thick) from the substrate using a lower‐bandgap selective 10 ns Nd:YAG laser pulse. The pulse selectivity and lattice matching enabled the removal of the multicrystalline layers, followed by etching and front‐contact deposition [188]. The resulting PV/fabrication device achieved 17.4% PCE under direct AM1.5 sunlight (1000 Wm^−2^). It is important to state explicitly that this is a solid‐state PV/fabrication result, not a PEC STH or applied‐bias photon‐to‐current efficiency (ABPE) value. While this physical separation can profoundly relieve lattice strain and exhibits excellent physical stability in a solar cell, there is no evidence that it serves as direct evidence of III−V PEC photoelectrode passivation. Future research providing insight into electrolyte interactions, applied‐bias performance, gas‐evolution tracking, etc., will be needed to validate the viability of PEC‐WS. Despite this, the physical fabrication strategy shows a very attractive route to strain‐relaxed III–V substrates.

**FIGURE 8:**
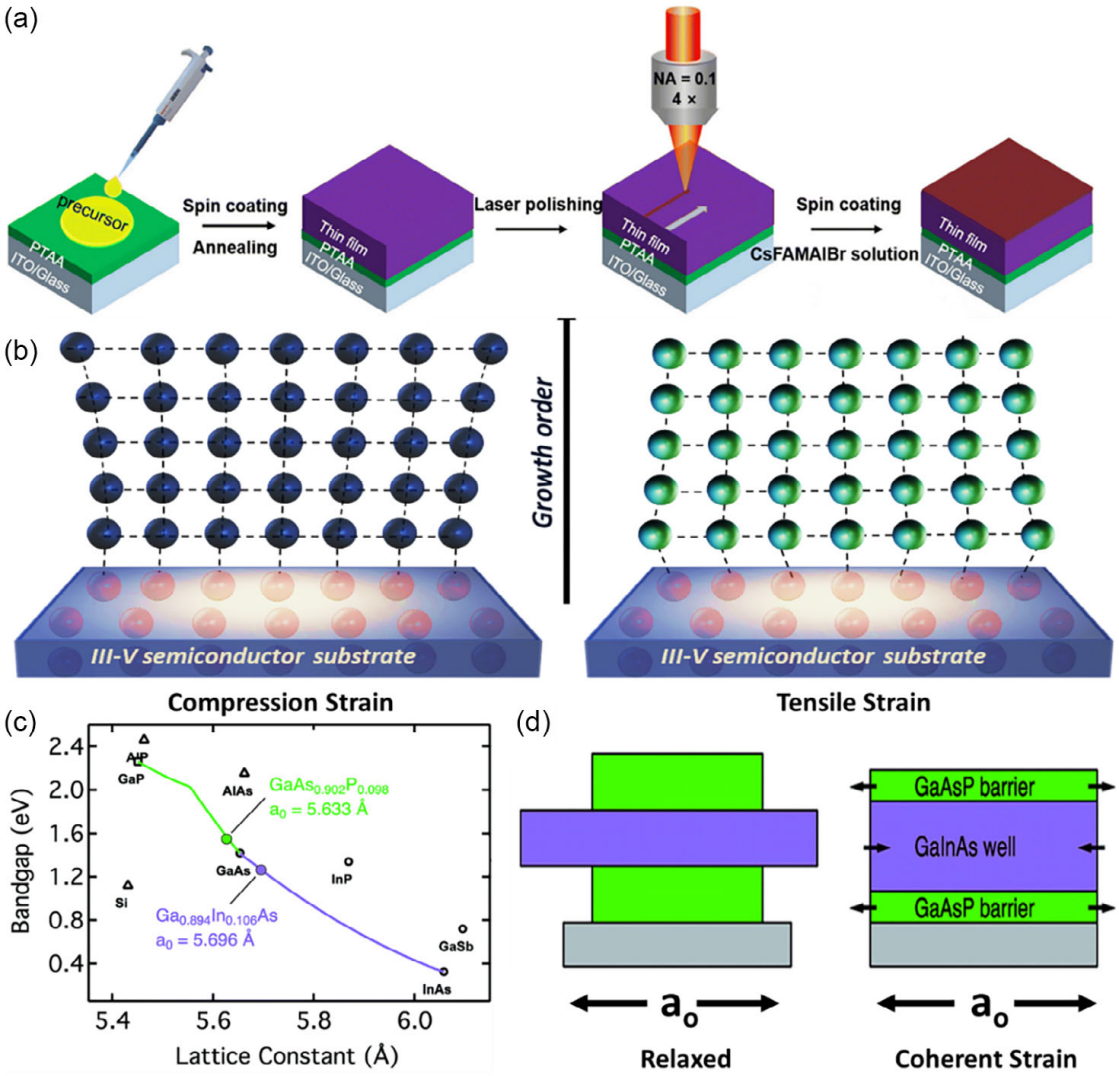
(a) Schematic representation of perovskite solar cell (PSC) fabrication process with femtosecond (fs) laser polishing. Reproduced with permission from ref. [185]. Copyright 2020, Wiley‐VCH. (b) Graphical illustration of compression and tensile strains on III–V substrate (red balls represent the lattice positions of the substrate atoms, while blue and green balls represent the atomic lattice positions of grown materials). (c) Bandgaps and lattice constants for common III–V binary and ternary alloys. Strain‐balanced GaInAs/GaAsP quantum wells with relaxed lattice constants are shown. (d) Relaxed and coherently strained layers of GaInAs (compressively strained) and GaAsP (tensile strained). Reproduced with permission from ref. [186]. Copyright 2019, Royal Society of Chemistry.

Strain relaxation of III–V photoelectrodes is another physical passivation method used to reduce surface defects [186]. By changing the growth rate the compressive or tensile strains can be controlled. (Figure 8b). Incorporation of strain‐balanced GaInAs/GaAsP quantum wells with 1.34 eV bandgap in the GaAs bottom junction achieved enhanced photon absorption up to 930 nm (Figure 8c). Due to the lattice mismatch between GaInAs and GaAsP alloys and GaAs, they need careful strain engineering to avoid the generation of dislocations that would affect the PEC‐WS photostability. Figure 8d shows strained and strain‐free electrodes. Tensile strain in GaAsP reduces the out‐of‐plane lattice constant, while compressive strain increases it. The strain‐balanced quantum well architecture improved the photostability and durability of PEC‐WS electrodes. Another study showed that InGaN NWs photocathodes are grown epitaxially on n‐type Si using a tunnel junction. The junction cut down interfacial dislocations and defects, allowing highly efficient charge transport assisted by defect‐free 1D NWs and photostability for about 300 h [189]. Although physical passivation enhances photostability, it requires precise control to optimize material composition. Table 3 shows a summary of the effect of surface passivation on the PEC‐WS performance of various III–V electrode materials. The comparative analysis reveals that passivation strategies not only significantly enhance device efficacy but also enhance photostability by increasing resistance to photocorrosion.

**TABLE 3:** Effect of surface passivation on the photostability and hydrogen generation efficiency of III–V photoelectrodes.

Photoelectrode composition	Passivation layer (thickness) @ deposition method	Electrolyte composition	PEC attributes of nonpassivated electrode	PEC attributes of passivated electrode	Ref.
GaAs NWs	TiO_2_ (7 nm) @ ALD	H_2_SO_4_ 0.5 M, pH = 1.0	GaAs NWs PL intensity = 13% after 14 months *I*_ph_ = 0.598 mAcm^−2^ @ 0 V_RHE_ *V*_onset_ = ∼0.243 V_RHE_ Photostability = 35 h FE, not given	GaAs NWs/TiO_2_ PL intensity = 91.4% after 14 months *I*_ph_ = 0.87 mAcm^−2^ @ 0 V_RHE_ *V*_onset_ = ∼0.233 V_RHE_ Photostability = 67 h FE = 91%	[134]
GaAs	TiO_2_ (4–143 nm) @ ALD and Ni (100 nm) @ sputtering	KOH 1.0 M, pH= 13.7	p^+^‐GaAs/TiO_2_/Ni‐film *I*_sc_ = 14.3 mAcm^−2^ @ 0.81 V_SCE_ *V*_onset_ = 0.81 V_SCE_ FE, not given Photostability = <25 h	p^+^‐GaAs/TiO_2_/Ni‐island *I*_sc_ = 37.1 mAcm^−2^ @ 0.81 V_SCE_ *V*_onset_ = 0.52 V_SCE_ FE = 99 ± 1% Photostability = 5% current drop in 100 h	[190]
GaAs	TiO_2_ (1–5 nm, best is 1 nm) @ ALD	H_2_SO_4_ 0.5 M, pH= 1.0	Bare GaAs *V*_onset_ = −0.05 V_RHE_ @ 1.0 mAcm^−2^ ABPE = 0.2%@ 0.2 V_RHE_ FE = 6%	GaAs/TiO_2_ *V*_onset_ = + 0.35 V_RHE_ @ 1.0 mAcm^−2^ ABPE = 1.5% @ 0.2 V_RHE_ FE = 92%	[191]
GaAs	TiO_2_ (3 nm) @ ALD	H_2_SO_4_, 0.5 M, pH = 1.0	Bare GaAs/Pt *V*_onset_ = −0.75 V_RHE_ @ 10 mAcm^−2^	GaAs/TiO_2_ (1 nm)/Pt *V*_onset_ = 0.32 V_RHE_ @ 10 Acm^−2^	[192]
GaAs	3D TiO_2_ (5 nm) and Ni particle @ GLAD	NaOH 1.0 M, pH =14	GaAs/TiO_2_ nanofilm *I*_ph_ = 8.43 mAcm^−2^ @ 1.0 V_RHE_ *V*_onset_ = −0.45 V_RHE_ IPCE = 38% Photostability = 62% loss @ 1 h	GaAs/TiO_2_/Ni top NRs *I*_ph_ = 10.42 mAcm^−2^ @ 0 V_Ag/AgCl_ *V*_onset_ = −0.53 V_Ag/AgCl_ (0.50 V_RHE_) IPCE = 52% Photostability = 56% loss @ 1h GaAs/TiO_2_/Ni coreshell NRs *I*_ph_ = 12.87 mAcm^−2^ @ 0 V_Ag/AgCl_ *V*_onset_ = −0.68 V_Ag/AgCl_ (0.35 V_RHE_) IPCE = 62% @ 0 V_Ag/AgCl_ (1 V_RHE_) Photostability = 15.6% loss in performance =1 h	[193]
Epitaxial GaAs	Pt (30 nm) @ sputtering	H_2_SO_4_ 0.5 M, pH = 1	Epitaxial GaAs *I*_sc_ = 21.8 mAcm^−2^ *V*_onset_ = ∼0.936 V_RHE_ Photostability = 38 h FE = 2.5%	Epitaxial GaAs/Pt *I*_sc_ = 22.5 mAcm^−2^ *V*_onset_ = ∼1.022 V_RHE_ Photostability = 70 h FE = 11.2%	[147]
InP NWs	S‐OA @ wet treatment	HCl 1.0 M, pH = 1.0	Planar InP *I*_sc_ = ∼22 mAcm^−2^ Photostability = 0.25 h (50% loss) ABPE = 3.9% @ 0.23 V_RHE_	InP NWs/Au treated with S‐OA *I*_sc_ = 33 mAcm^−2^ Photostability = 10 h (∼5% loss) ABPE = 5.2% @ 0.23 V_RHE_	[194]
p‐InP nanopillars (NPLs)	TiO_2_ (3–5 nm) @ ALD and a Ru cocatalyst @ sputtering	HClO_4_ 1.0 M, pH = 0.5	Ru ‐ TiO_2_ @ planar p‐InP *I*_sc_ = ∼27 mAcm^−2^ @ 0.5 V_NHE_ *V*_onset_ = 0.5 V_NHE_ Photostability = 2 h (50% *I*_ph_ loss) H_2_ production efficiency = ∼9%	Ru‐TiO_2_ @ NPLs p‐InP *I*_sc_ = ∼37 mAcm^−2^ @ 0.73 V_NHE_ *V*_onset_ = 0.73 V_NHE_ Photostability = 4 h (< 5% *I*_ph_ loss) H_2_ production efficiency = ∼14%	[49]
p‐InP	TiO_2_ (10 nm) @ ALD	HClO_4_ 1.0 M, pH 0.3	InP photocathode *I*_sc_ = 24 mAcm^−2^ *V*_onset_ = ~0.63 V_RHE_ IPCE = <30% @ 0.2 V_RHE_	p‐InP/TiO_2_ Photocathodes *I*_sc_ = 25.5 mAcm^−2^ @ V_RHE_ *V*_onset_ = ~0.81 V_RHE_ IPCE = 70‐80% @ 0.2 V_RHE_ FE = 95%	[195]
GaInP_2_	MoS_2_ film @ partial thermal sulfidization	H_2_SO_4_ 3.0 M, pH = 1.0	pn^+^‐GaInP_2_/PtRu *I*_sc_ = ∼15 mAcm^−2^ *V*_onset_ = 0.83 V_RHE_Photostability = ∼6 h	pn^+^‐GaInP_2_/MoS_2_ *I*_sc_ = ∼13 mAcm^−2^ *V*_onset_ = 0.91 V_RHE_ @ 1 mAcm^−2^ Photostability = 100 h	[180]
p‐GaInP_2_	TiO_2_ (35 nm) @ ALD, functionalized by cobaloxime (GaInP_2_/TiO_2_/cobaloxime)	NaOH 0.1 M, pH = 13	Bare p‐GaInP_2_ *I*_ph_ = ∼0.3 mAcm^−2^ @ 0 V_RHE_ *I*_sc_ = ∼11 mAcm^−2^ @ 1.05 V_Ag/AgCl_ Photostability = ≤5% IPCE = ∼45%	GaInP_2_/TiO_2_/cobaloxime *I*_ph_ = ∼9 mAcm^−2^ @ 0 V_RHE_ *I*_sc_ = ∼13 mAcm^−2^ @ 1.05 V_Ag/AgCl_ Photostability = ≤5% photocurrent decrease during the 20 min IPCE = ∼80%	[104]
InGaAsP	TiO_2_ (26 nm) @ ALD	HCl 1.0 M, pH = 1.0	Bare InGaAsP *I*_sc_ = 20 mAcm^−2^ *V*_sat_ = 0.25 V_RHE_ ABPE = 1% Photostability = 50% current drop in 3 min	InGaAsP/TiO_2_ *I*_sc_ = 30 mAcm^−2^ *V*_sat_ = 0.20 V_RHE_ ABPE = 7%Photostability = 38% current drop in 3.5 h	[196]
InGaN/GaN multiple quantum well (MQW)	CoO_*x*_ (12–13 nm) @ ALD	NaOH 1.0 M, pH = 13.7	InGaN/GaN MQW *I*_ph_ = 0.94 mAcm^−2^ @ 1.23 V_RHE_ *V*_onset_ = 0.01 V @ 1.23 V_RHE_ IPCE = ∼10.3% @ 380 nm Photostability = 40 min	InGaN/GaN MQW‐CoO_*x*_ *I*_ph_ = 1.26 mAcm^−2^ @ 1.23 V_RHE_ *V*_onset_ = −0.03 @ 1.23 V_RHE_ IPCE = 16.8% @ 380 nm Photostability = 28 h	[197]
Single‐junction Ga‐assisted GaAs_0.8_P_0.2_ NWs	InGaP (10 nm) @ annealing at high temperature	NA	Ga‐GaAs_0.8_P_0.2_ NWs *I*_sc_ = 13.2 mAcm^−2^ *V*_oc_ = 0.76 V FE = 6.8% ff = 68%	Ga‐GaAs_0.8_P_0.2_ NWs/InGaP *I*_sc_ = 14.7 mAcm^−2^ *V*_oc_ = 0.9 V FE = 10.2% ff = 77%	[198]
Dual‐junction tandem GaInP/GaInAs	TiO_2_ (30 nm) @ ALD	KH_2_PO_4_/K_2_HPO_4_ phosphate buffer, 0.5 M, pH = 7.0	GaAs/GaInAs/GaInP/AlInP *I*_ph_ = not given *η*_STH_ = 15.1% Photostability = <2 h Contact angle = 68.3°	GaAs/GaInAs/GaInP/AlInP–TiO_2_ (30 nm) *I*_ph_ = 13.17 mAcm^−2^ @ 0 $V_{{\mathrm{RuO}}_{2}}$ *η*_STH_ = 19.3% Photostability = ~20 h Contact angle = 24.5°	[45]

Abbreviations: ALD, atomic layer deposition; GLAD, glancing angle deposition; S‐OA, sulfur‐dissolved oleylamine.

Although both intrinsic passivation and extrinsic passivation work, using them in an industrial PEC‐WS is more difficult than successfully executing them in the laboratory. The main challenge lies in the delicate “thickness‐conductivity trade‐off” in ALD‐deposited metal oxides. Layers must be thick enough to suppress pinholes and prevent the ingress of corrosive ions, but thin enough to allow quantum tunneling or direct charge transfer without incurring detrimental resistive losses. Furthermore, the vast combinatorial space of possible passivation materials from different ternary oxides and complex organic functional groups renders empirical trial‐and‐error discovery extremely inefficient. In the future, the success of high‐throughput computational screening and machine learning models will be crucial. Through the utilization of advanced machine learning software, within the context of materials informatics, it will be able to predict valence and conduction band alignments rapidly between a new passivation layer and a relevant underlying III–V substrate. At the same time, thermodynamic phase stability simulations in strongly acidic or alkaline environments can also be achieved. By integrating such predictive models with automated robotic synthesis platforms, the process of discovering optimal, defect‐free passivation architectures can be expedited.

The adoption of ML and generative AI as a new model for the intrinsic and extrinsic surface passivation of III–V semiconductors has shifted the approach from empirical trial and error to a data‐driven basis for predictions. A critical overview shows transformative potential without limitations. ML models, particularly neural networks and Gaussian process regression, have successfully mapped complex processing spaces, such as temperature, time, and precursor concentration, to minimize surface state density [156, 199, 200]. For example, by using the spectroscopic data, the prediction of optimum arsenic‐sulfur stoichiometry by the AI is faster than the trial‐and‐error process [201]. The important pitfall is the lack of data. High‐quality surface science datasets are small and often instrument‐specific, making models prone to overfitting and poor generalizability across different crystal orientations or doping levels.

Various algorithm models are reported for optimizing the passivation for successful photocorrosion inhibition, as described in the literature [202, 203, 204, 205]. For example, the gradient‐boosted passivity classifier (XGBoost) was used to classify passivity levels in alloy systems, identify dominant frequency bands, and provide interpretable links between passivation and inhibitor effects [201]. The random forest (RF) pitting‐profiling models predicted pitting‐rate evolution and captured passivation breakdown in steel inhibitor media, thereby improving understanding of inhibitor‐assisted re‐passivation mechanisms [101, 201, 206]. Deep learning algorithms applied to SEM and AFM images quantify pit density, film uniformity, and the evolution of surface roughness across different inhibitor concentrations [207]. These image features serve as additional ML descriptors linking microstructural evolution to inhibition efficiency. Convolutional Neural Networks integrated with EIS data achieved >90% classification accuracy in distinguishing protected versus unprotected surfaces, enabling automated surface feature classification [208]. As a case study, a SMILES‐X classifier trained on literature data achieved high predictive performance (F1 = 0.80, ROC–AUC = 0.88), while a GPT‐2‐based generative model iteratively produced over 100, 000 novel molecules, more than 80% of which were predicted to be effective in photo‐driven solar cell applications [181].

These approaches represent a significant advancement in automated passivation analysis, shifting from manual interpretation to AI‐driven surface‐protection assessment. However, the “black‐box” nature of deep learning obscures physical passivation mechanisms, e.g., the distinction between true chemical passivation and field‐effect passivation. Most models treat surface chemistry as a static feature, ignoring dynamic degradation under operating conditions (light, bias, temperature). Furthermore, transfer learning from oxide‐free III–V surfaces to real‐world, air‐exposed devices remains unreliable due to native oxide heterogeneity.

Importantly, both intrinsic and extrinsic approaches in ML literature suffer from a lack of unified descriptors. While AI can correlate process parameters with electrical outputs (photoluminescence intensity), it rarely incorporates atomistic simulations (DFT) to rationalize why a particular passivation works. The most robust path forward is hybrid modeling that uses ML to navigate high‐dimensional process space while constrained by physics‐based insights on bond coordination and band alignment. Without this integration, AI risks becoming a curve‐fitting exercise rather than a genuine tool for understanding III–V surface science [209].

### 4.3 Heterostructure Formation of III–V Semiconductors

Modifying the bulk composition of parent III–V semiconductors via nanocomposite or dopant heterostructure formation presents an attractive strategy to enhance the stability of the PEC‐WS process against photocorrosion. Metallic and nonmetallic doping of III–V semiconductors has been extensively explored for decades [210, 211].

First‐principles calculations on p‐ and n‐type dopant formation energies in III–V arsenide and phosphide semiconductors reveal that dopant level shallowness and solubility depend on the wurtzite or zinc blende crystal phase of the host material. These calculations suggest that p‐type doping is more easily achieved in a wurtzite crystal structure [212]. In this context, metal dopants are particularly effective in enhancing the PEC behavior of parent III–V semiconductors. The density and free‐carrier type (n‐ or p‐type) of III–V semiconductor films can be adjusted by incorporating metallic impurities, such as In, Te, Zn, Sb, and Si, during growth [48]. For example, modifying the composition of GaP by adding 3% metallic Sb to form a GaSb_0.03_P_0.97_ trimetallic alloy enhances the absorption of high‐energy photons (>2.68 eV) compared with pure GaP. Moreover, doping with silicon changes the carrier nature of the n‐GaSb_0.03_P_0.97_ semiconductor, leading to a high photocurrent density during PEC‐WS [31]. Similarly, a p‐type InP nanotextured photocathode demonstrated a significant photocurrent density of 37 mA cm^−2^ at 0.73 V_NHE_, with STH efficiency of 14% [49]. Epitaxially grown n‐type GaN/In_*x*_Ga_1–*x*_N core–shell rod arrays show promise for PEC‐WS, with varying Indium (In) concentrations (*x* ≈ 0.3). The rod array morphology plays a crucial role by enabling the homogeneous incorporation of indium, which helps mitigate dislocations and strain [213]. Numerical simulations show that the depletion depths in the p‐type modification layer and n‐type photon absorber must be optimized by dopant densities to maximize the performance of the p–n junction in a PEC‐WS device [33].

In the early 2000s, nonmetallic dopants, particularly boron‐doped III–V semiconductors, were investigated, with a primary focus on optimizing growth conditions. Boron doping (acting as a small‐radius dopant) was introduced into InGaAs layers to form BGaInAs alloys with a 4% boron concentration, effectively lattice‐matching the GaAs substrate [214]. Lattice matching enhanced the photoactivity of the device by reducing strain and minimizing surface defects. A core–shell boron‐doped heterojunction, a B_*x*_Ga_1−*x*_As/GaAs photocatalyst, exhibited controllable boron dopants, resulting in a high‐quality heterojunction interface (Figure 9a) [255]. This tunable heterostructure produced 8.4 μmol of hydrogen after 8 h of irradiation. High‐temperature phosphidation of a GaN solid solution also significantly enhanced PEC‐WS performance when the *x* range in GaP_1−*x*_N_*x*_ was 0.034–0.074 [216]. Nitrogen doping is another well‐established method to ameliorate the photocatalytic properties of many semiconductors. In indirect‐gap III–V semiconductors, such as GaP, nitrogen impurities introduce bound states that significantly enhance absorption below the indirect bandgap, effectively reducing the absorption edge energy. Conversely, nitrogen impurities in direct‐band‐gap III–V semiconductors, such as GaAs, form resonant states above the conduction band edge. A similar effect is observed in phosphorus‐doped GaAs semiconductors, but nitrogen is more commonly used to dilute III–V alloys than as a traditional dopant. Therefore, its use as a dopant in III–V materials remains limited and has not been extensively explored for PEC‐WS applications [217].

**FIGURE 9:**
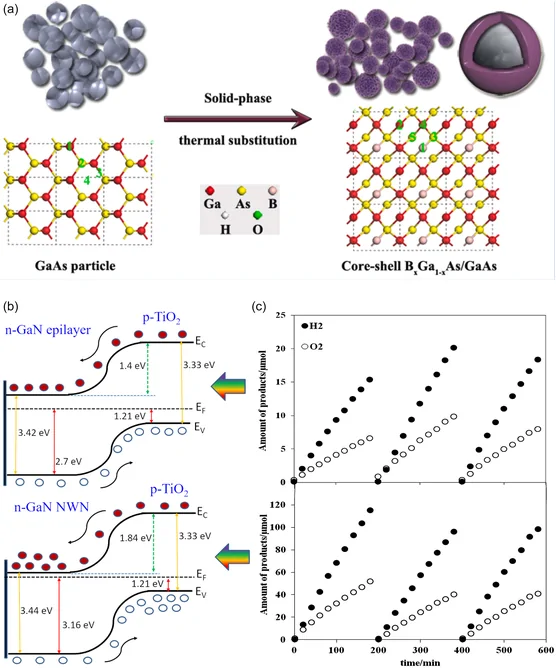
(a) Schematic representation of core–shell B_*x*_Ga_1−*x*_As/GaAs photocatalysts synthesis. Reproduced with permission from ref. [255]. Copyright 2021, Elsevier. (b) Band diagrams for TiO_2_/GaN epilayer (left) and TiO_2_/GaN nanowall network (NWN) (right) heterostructures. Reproduced with permission from ref. [215]. Copyright 2018, IOP Publishing. (c) Evolution of H_2_ and O_2_ during water splitting by untreated ZnO/GaN (left) and phosphatized ZnO/GaN‐P (right). Filled circles represent H_2_, and open circles represent O_2_. Reproduced with permission from ref. [216]. Copyright 2015, Royal Society of Chemistry.

Nanocomposite formation with III–V semiconductors is an effective strategy for enhancing PEC‐WS performance. InP NPs and quantum dots (QDs) are well‐established III–V complementary photocatalysts for PEC‐HER. DFT calculations on cage‐like InP QDs sensitized with TiO_2_ using first‐principle molecular dynamics simulations demonstrated a bandgap reduction to 2.0 eV. The optoelectrical calculations revealed a type‐II band alignment, which helps suppress recombination reactions and enhance PEC‐WS performance [218]. For instance, the sulfonate‐functionalized ligands have modified InP/ZnSe (core/shell) QDs. The reduction of the band‐edge PL intensity of the TiO_2_ NPs and sulfonate‐treated InP/ZnSe composites promoted the transfer of photoexcited electrons to TiO_2_. These spatially separated photogenerated charge carriers enhance the photocatalytic efficiency of InP/ZnSe QD–TiO_2_ composites by effectively suppressing charge recombination [219]. Similarly, reduced charge–transfer resistance and increased band bending at the MoS_*x*_/Ti/InP/electrolyte interface result in a significant PEC‐WS photocurrent of 15.8 mA cm^−2^ at 0.62 V _RHE,_ but photostability is limited to only 2 h [220]. However, replacing MoS with ZnS significantly enhanced photostability to 40 h for Cu‐doped InP/ZnS (InP/Cu:ZnS) nanocomposites. This enhancement is attributed to the more robust ZnS shell, which provided supplementary mechanical stability, and the addition of a benzoquinone electron acceptor that effectively suppressed electron–hole recombination [221].

GaN‐based III–V photocatalysts are predominantly utilized for overall water splitting. In a pioneering study, a RuO_2_–GaN/ZnO nanocomposite with a bandgap of 2.58–2.76 eV demonstrated successful overall water splitting. This was followed by the extensive utilization of GaN for PEC‐WS application [222]. In one study, a p‐TiO_2_/n‐GaN heterostructure was fabricated in two steps. First, the GaN was grown in its wurtzite form by MBE, resulting in two morphologies: a GaN nanowall network (TiO_2_/GaN NWN) and a planar GaN heterostructure (TiO_2_/GaN epilayer). The TiO_2_ overlayer was deposited using ALD. The TiO_2_/GaN NWN photocurrent density was approximately 0.65 mA cm^−2^ with 17% IPCE, compared with 0.24 mA cm^−2^ and 6% IPCE for the TiO_2_/GaN epilayer under 13 mW cm^−2^ solar power. Chronoamperometric analysis shows that the electrodes exhibited high stability. The resulting p–n diagram reveals that the TiO_2_/GaN NWN exhibited a higher barrier height arising from interfacial band bending, which promoted superior charge separation (Figure 9b) [215]. A slight modification of GaN/ZnO nanocolumns with NiO resulted in a 2.1‐fold increase in light absorption compared with that in GaN nanocolumns. This enhancement is attributed to the formation of a heterojunction between GaN and ZnO, as well as a p–n junction between ZnO and NiO. Furthermore, the lower refractive indices of NiO and ZnO than those of GaN also facilitated light absorption in the composite [223]. GaP was also utilized for PEC‐HER activity as a photocathode; nonetheless, its selective chemical modification can also enable overall water splitting. For instance, RuO_2_–ZnO/GaN and RuO_2_–ZnO/GaN‐P nanocomposites demonstrated immediate H_2_ and O_2_ production under illumination, with a 2.2:1.0 production ratio, respectively. Photostability was maintained up to the fourth cycle (Figure 9c) [216]. Finally, the comparative activity of RuO_2_–ZnO/GaN‐P nanocomposites was nearly eight times higher than that of RuO_2_–ZnO/GaN, but modifications with foreign materials often introduce structural and functional complexities in III–V semiconductors, while also increasing the production cost of PEC‐WS devices.

Despite decades of extensive research efforts, none of the heterostructure formation strategies is commercially viable, and researchers are seeking additional alternatives. Lately, ML‐enhanced dopant selection methods have been employed in various PEC systems to reduce the number of experimental trials and predict optimal modifying species [224, 225]. For instance, Wang et al. successfully built ML models to predict the doping effects of 17 metal dopants in hematite (Fe_2_O_3_), a prototype photoelectrode material, and further validated them experimentally [226]. The interpretation of the ML model suggests that chemical state is the most significant selection criterion. Meanwhile, dopants with higher metal–oxygen bond formation enthalpies and larger ionic radii are favored for improving charge separation and transfer (CST) in Fe_2_O_3_ photoanodes.

The generic feature of this ML‐guided selection criterion has been further extended to CuO‐based photoelectrodes, showing improved CST upon alkaline metal ion exposure [226]. The schematic of the ML model used for this study is shown in Figure 10a, while the respective outcomes are given in Figure 10b–h. A framework was developed to investigate the influence of dopants based on their intrinsic structure features (database S). Database S consists of 11 descriptors, including atomic number (*N*), ionic radius (*r* *_i_*), atomic radius (*r*_a_), molecular single bond covalent radius (*r*_c_), chemical valence (*Z*), M─O bond formation enthalpy (Δ*f*_H_) based on metal (Δ*f*_H‐M_) and oxygen (Δ*f*_H‐O_) respectively, electronegativity (*χ*), electron affinity (*A*) of elements, melting point of pure metal (*T*_m_), and dopant concentration (*c*). In addition, the performance of the photoelectrode (database P) was evaluated based on the plateau photocurrent density measured in NaOH solution for experimental comparison. These models can be extended to PEC‐WS systems based on III–V photoelectrodes. However, the lack of III–V‐specific literature and dataset resources makes their practical implications challenging in the near future.

**FIGURE 10:**
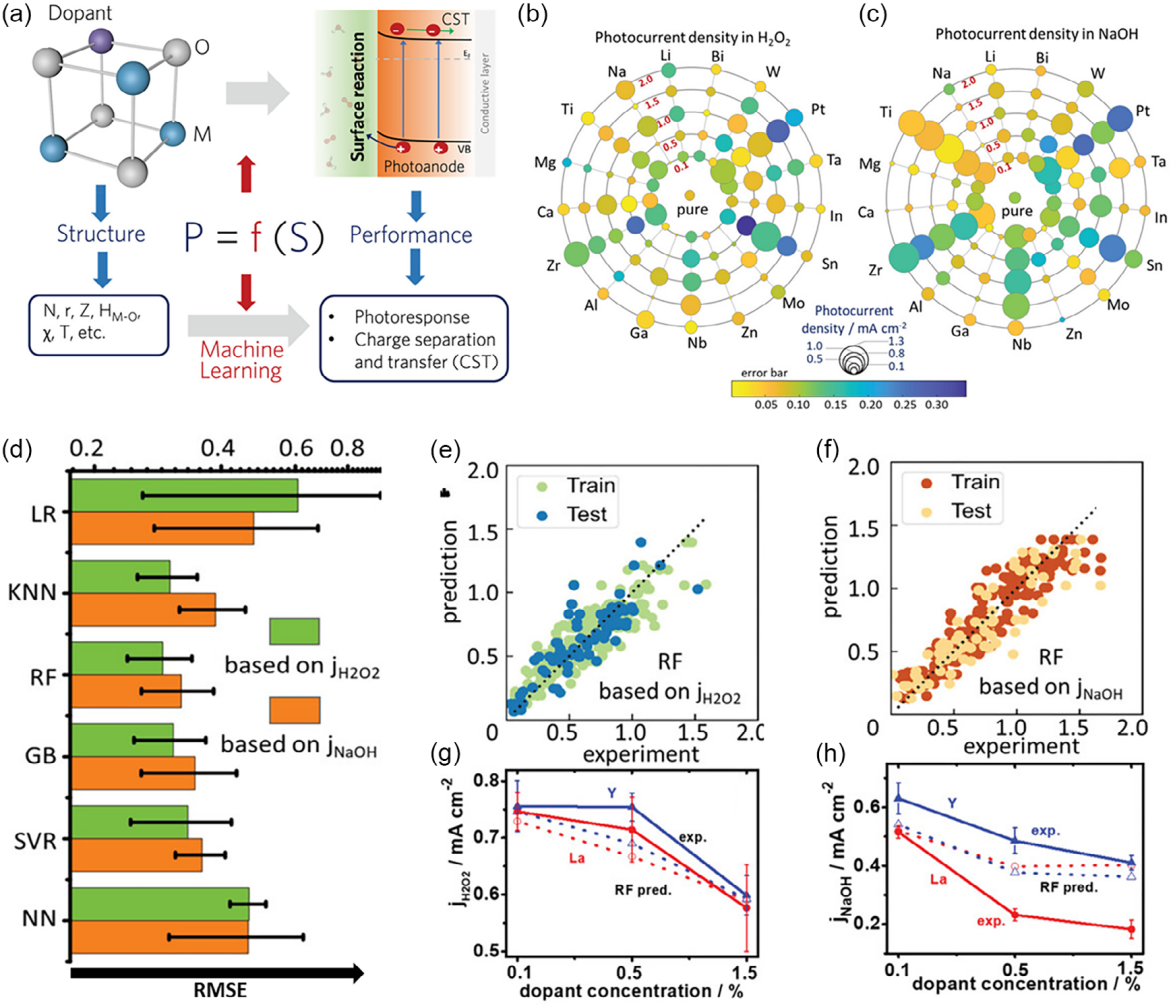
(a) The schematic illustration of machine learning‐guided dopant selection toward efficient PEC process. (b, c) Comparison of photocurrent density between pure and the doped Fe_2_O_3_ photoanodes in the presence of 0.2 mol L^−1^ H_2_O_2_ as hole scavenger and in 1 mol L^−1^ NaOH solution. The circle's diameter quantifies the magnitude of photocurrent density, and the measurement errors are indicated by the colored error bar. Dopant concentration taken in %. (d) Model evaluation for five ML models based on RMSE for predicting $j_{H_{2}O_{2}}$ and *j*_NaOH,_ respectively_._ (e,f) Training and test results using RF model for $j_{H_{2}O_{2}}$ and *j*_NaOH,_ respectively. (g, h) Comparison of predicted (dotted lines) and experimental (solid lines) $j_{H_{2}O_{2}}$ and *j*_NaOH_ with error bars for La and Y dopants Fe_2_O_3_ photoelectrode. Reproduced with permission from ref. [226]. Copyright 2022, Wiley‐VCH.

Moreover, ML algorithms perform well on physical interactions but face significant drawbacks for chemical interactions due to various unmeasurable complexities. One solution to overcome these challenges is to combine all available datasets with experimental datasets in hybrid ML‐enhanced methods [200, 224, 225]. Table 4 compares the PEC‐WS performance of pristine III–V photoelectrodes, their heterostructures, and nanocomposites. The findings clearly demonstrate that modifications significantly enhance the performance of bare or pristine III–V electrodes, owing to the formation of suitable Type I or Type II heterojunctions, which facilitate charge transfer mechanisms [84, 85].

**TABLE 4:** Comparative PEC‐WS attributes for bare and nanocomposites III–V photoelectrodes.

Photoelectrode composition	Electrolyte composition	PEC attributes of bare electrode	PEC attributes of the modified electrode	Ref.
Printed GaAs with Pt (30 nm cocatalyst)	H_2_SO_4_ 0.5 M, pH = 1	Printed GaAs *I*_sc_ = 21.8 mAcm^−2^ @ −0.25 V_RHE_ *V*_onset_ = ∼0.936 V_RHE_ Photostability = 38 h *η*_STH_ = not given	Printed GaAs with Pt (30 nm cocatalyst) *I*_sc_ = 22.5 mAcm^−2^ @ −0.77 V_RHE_ *V*_onset_ = ∼1.022 V_RHE_ Photostability = 70 h *η*_STH_ = 13.1%	[147]
Au/GaAs	KCl 1 M, pH = 7	GaAs metasurface *I*_sc_ = 38 mAcm^−2^ @ 730 nm *I*_sc_ = 0.7 mAcm^−2^ @ white light	ms‐Au/GaAs *I*_sc_ = 113 mAcm^−2^ @ 730 nm *I*_sc_ = 2.2 mAcm^−2^ @ white light	[227]
Pt@InP NPAs	Na_2_S (0.35 M) Na_2_SO_3_ (0.5 M) pH = 13.6	Pure InP photoanode *I*_sat_ = 0.62 mAcm^−2^ @ −0.8 V_Ag/AgCl_ *V*_onset_ = −0.77 V_Ag/AgCl_	Pt@InP NPAs *I*_sat_ = 3.44 mAcm^−2^ @ −0.8 V_Ag/AgCl_ *V*_onset_ = −0.92 V_Ag/AgCl_	[228]
p‐InP single crystal modified by Co and Pt	KOH 6 N pH = 14.0	p‐InP electrode *I*_ph_ = 4 mAcm^−2^ @ −0.8 V_SCE_ *V*_oc_ = −0.35 V_SCE_ FF = 0.23	InP/Co *I*_ph_ = 13 mAcm^−2^ @ −0.8 V_SCE_ *V*_oc_ = −300 mV FF = 0.48 InP/Co/Pt *I*_ph_ = 14 mAcm^−2^ @ −0.8 V_SCE_ *V*_oc_ = −200 mV_SCE_ FF = 0.48	[229]
[p‐type InP(Ru)]/HCl‐KCl/Pt(Rh)	HCl 1 M and KCl 2 M	p‐type InP *I*_ph_ = 20 mAcm^−2^ @ 0.31 V_SCE_ *V*_onset_ = −0.24 V_SCE_ *η*_STH_ = not given	p‐type InP with Pt *I*_ph_ = 20 mAcm^−2^ @ 0.15 V_SCE_ *V*_onset_ = −0.34 V_SCE_ *η*_STH_ = 12%	[103]
Au@InP NPAs	Na_2_S (0.35 M) Na_2_SO_3_ (0.5 M) pH = 13.6	Pure InP NPAs *I*_ph_ = ~2.8 mAcm^−2^ @ −0.7 V_Ag/AgCl_ *V*_onset_ = −0.80 V_Ag/AgCl_	Au@InP NPAs *I*_ph_ = 10.2 mAcm^−2^ *V*_onset_ = −0.95 V_Ag/AgCl_	[230]
In_30_Ga_70_N NWs/Si (111)	NaOH 1 M pH = 14	GaN NWs/Si *I*_max_(sat) = 1.52 mAcm^−2^ @ 0.5 V_RHE_ *V*_onset_ = −0.31 V IPCE = 7.2% ABPE = 1.1%	In_30_Ga_70_N NWs/Si (111) *I*_max_(sat) = 8.08 mAcm^−2^ @ 0.5 V_RHE_ *V*_onset_ = −1.04 V IPCE = 19.1% @ 520 nm ABPE = 5.8% H_2_ rate = 42.3 μmol cm^−2^ h^−1^ Photostability = 3.5 h	[189]
InGaN/MXene	H_2_SO_4_ 0.5 M pH = 0.3	InGaN/Si photoanode *I*_ph_ = 0.71 mAcm^−2^ @ 1.23 V_RHE_ ABPE = 0.24% @ 0.73 V_RHE_Photocurrent decay = 76%	InGaN NRs/MXene/Si Photoanode *I*_ph_ = 7.27 mAcm^−2^ @ 1.23 V_RHE_ ABPE = 2.36% @ 0.63 V_RHE_ Photocurrent decay = remains 35% @ 1 h	[231]
InGaN/GaN MQW	NaOH 1 M pH = 14	GaN reference photoanode *I*_ph_ = 0.48 mAcm^−2^ *V*_onset_ = ∼−1.1 V Planar photoanode Photo absorption = 370 nm	InGaN/GaN MQW (In = 30%) *I*_ph_ = 1.6 mAcm^−2^ InGaN/GaN MQW (In = 50%) *I*_ph_ = 0.4 mAcm^−2^ *V*_onset_ = ∼−1.0 V Photo absorption = 450 nm	[232]
Pin‐AlGaAs (TiO_2_ 60 nm)	HCl 1 M pH = 0	p‐AlGaAs *I*_sc_ = 15.0 mAcm^−2^ *V*_onset_ = 0.80 V_RHE_ *η*_STH_ = 2.5%	TiO_2_ covered pin‐AlGaAs *I*_sc_ = 15.1 mAcm^−2^ *V*_onset_ = 1.11 V_RHE_ IPCE = 85% at 675 nm *η*_HC_ = 8.9%	[233]
Dual‐junction tandem GaInP/GaInAs (TiO_2_ 30 nm) photoelectrode	KH_2_PO_4_/K_2_HPO_4_ phosphate buffer, 0.5 M, pH = 7.0	GaAs/GaInAs/GaInP/AlInP *I*_ph_ = not given *η*_STH_ = 15.1% Photostability = <2 h Contact angle = 68.3°	GaAs/GaInAs/GaInP/AlInP‐TiO_2_ (30 nm) *I*_ph_ = 13.17 mAcm^−2^ @ 0 $V_{{\mathrm{RuO}}_{2}}$ *η*_STH_ = 19.3% Photostability = ~20 h Contact angle = 24.5°	[45]
NiO_*x*_ @ n‐GaN (NiO_*x*_ = 1:5 and 3:2)	1 M NaCl pH = 6	n‐GaN photoanode *I*_WE vs. CE_ = 1.15 mAcm^−2^ @ 1 V_WE vs. CE_ *V*_onset_ = 0.60 V_WE vs. CE_ *V*_ph_ = 0.37 V IPCE, ~20% ABPE = 0.05% at 0.90 V	NiO*_x_* over n‐GaN NiO*_x_* (1:5) over n‐GaN *I*_WE vs. CE_ = 1.59 mAcm^−2^ @ 1.0 V_WE vs. CE_ *V*_onset_ = 0.30 V_WE vs. CE_ *V*_ph_ = 0.47 V IPCE, ~35% ABPE = 0.08% at 0.76 V PEC stability = 5 h @ 1 V	[234]
			NiO*_x_* (3:2) over n‐GaN *I*_WE vs. CE_ = 1.71 mAcm^−2^ @ 1 V_WE vs. CE_ *V*_onset_ = 0.30 V_WE vs. CE_ *V*_ph_ = 0.53 V IPCE, ~40% ABPE = 0.10% at 0.76 V PEC stability = 5 h @ 1 V (higher than 1:5)	
FeOOH/n‐GaN composite films	1 M NaCl pH = 6.1	Bare n‐GaN *I*_ph_ = 0.067 mAcm^−2^ *V*_fb_ = −1.63 V *R*_S_ = 18.3 Ω H_2_ rate = 1.19 µmolcm^−2^h^−1^	FeOOH, decorated GaN photoelectrodes *I*_ph_ = 0.316 mAcm^−2^ *V*_fb_ = −1.73 V *R*_S_ = 15.7 Ω H_2_ rate = 4.89 µmolcm^−2^h^−1^	[235]

*Note*: Potentials are reported vs. RHE wherever feasible. Where the original literature omitted sufficient calibration or pH data required for accurate Nernstian conversion, potentials are retained in their originally reported reference scale (SCE, Ag/AgCl) to preserve data integrity.

Abbreviations: Au@InP NPAs, Au decorated InP nanopore arrays; MQW, multiple quantum well; NRs, nanorods; NWs, nanowires; Pt@InP NPAs, Pt decorated InP nanopore arrays.

The most important future unsolved question is how to translate these decoupled, ML‐assisted dopant and heterostructure predictions into commercially relevant III–V PEC‐WS architectures. The main obstacle is the severe scarcity of high‐fidelity, standardized datasets on dopant dynamics, interfacial defect density, and lattice‐mismatch strain in ternary and quaternary III‐Vs. Although any metal–oxide proxy model (for instance, Fe_2_O_3_) could provide useful first‐principles insights, III–V semiconductors possess fundamentally different covalent bonding character and surface‐state dynamics that require domain‐specific training data. In addition, to bridge the gap between computational prediction and laboratory synthesis, we must move from basic descriptive models to generative AI systems that enable high‐throughput autonomous screening. Future breakthroughs will require “closed‐loop” autonomous laboratories in which AI algorithms will suggest new multi‐junction III–V heterostructures and iteratively control robotic synthesis and operando electrochemical characterization, allowing for dynamic learning from chemical feedback, leading to optimally stable and defect‐free photoelectrodes.

## 5 Current Status of AI/ML for III–V Photoelectrodes

The integration of artificial intelligence and machine learning into PEC‐WS research offered considerable excitement, yet a critical examination of the literature reveals a striking disparity between the promise of these approaches and their actual demonstration for III–V semiconductor systems. The studies summarized in Table 5 illustrate this disconnect with remarkable clarity, exposing a field that remains largely speculative when it comes to III–V PEC applications. A clear distinction between AI/ML demonstrated directly for (i) III–V PEC systems, (ii) III–V processing but not PEC operation, and (iii) methods extrapolated from proxy systems is necessary to understand the true implications of these tools.

**TABLE 5:** Summary of computational and AI/ML studies applied to III–V semiconductors and related transferable analogies.

Optimization task	AI/ML method	Input descriptors	Material system	Direct III–V PEC vs. transferable analogy	Primary limitation	References
Crystal growth and morphology control	Resnet‐50 (deep learning)	In situ RHEED video frames, substrate temperature	InAs/GaAs quantum dots	Direct III–V evidence	High cost of in situ monitoring equipment, black‐box nature obscures kinetic mechanisms	[114, 126]
Crystal structure and phase prediction	Generative AI (denoising diffusion/GNNs)	Textual descriptions, 3D structural data	General inorganic crystals	Transferable analogy	Predictions heavily rely on existing ICSD data, struggle with transient/metastable phases	[157]
Passivation layer selection	Random Forest/XGBoost	Electronegativity, bond enthalpy, molecular SMILES	Perovskites/Aluminum alloys	Transferable analogy	Fails to account for domain‐specific III–V native oxide heterogeneity and highly acidic/alkaline dynamic degradation	[209, 236]
Band‐gap and alignment engineering	Neural networks/ridge regression	Stoichiometry, strain percentage	Quaternary III–V (GaAsPSb)	Direct III–V evidence	Lacks comprehensive data on hexagonal phase III–V semiconductors and deep‐level capture rates	[237]
Dopant screening and CST prediction	LASSO Regression/Random Forest	Atomic/ionic radius, valence, M─O bond formation enthalpy	Metal Oxides (Fe_2_O_3_, CuO)	Transferable analogy	Covalent bonding character and surface‐state dynamics of III‐Vs differ fundamentally from oxides	[226, 238]
Electrochemical interface modeling	Ab initio molecular dynamics (ElectroFace Dataset)	Atomic coordinates, explicitly solvated interfaces	General electrochemical interfaces	Transferable analogy	Computationally expensive to simulate long‐term anodic photocorrosion kinetics	[79]
Defect/abnormal grain prediction	LSTM/graph convolutional recurrent networks	Grain volumes, boundary velocity, integral curvature	Polycrystalline metals/ceramics	Transferable analogy	Requires high‐fidelity	[159]
Charge transport and impedance analysis	Support vector regression	Modulation frequency (for Nyquist trajectory reconstruction)	n‐type GaAs	Direct III–V evidence	Strictly constrained to the measured frequency window	[239]
Temperature‐dependent defect and electronic structure prediction	Equivariant graph neural networks (MACE + DeepH‐E3 in active learning)	Atomic coordinates and thermal vibrational dynamics	Defective GaAs (charge‐neutral point defects like ${\mathrm{As}}_{\mathrm{Ga}}$)	Direct III–V evidence	Currently restricted to charge‐neutral defects and relying on semilocal DFT	[240]
Cost‐Aware mechanical characterization and property mapping	Cost‐aware Bayesian optimization with heteroskedastic Gaussian processes	Spatial coordinates mapping to composition gradients, local measurement noise, and time/drift costs	Combinatorial thin‐film libraries (Ta‐Ti‐Hf‐Zr alloys)	Transferable analogy	Susceptible to kernel‐length scale collapse in highly discontinuous or noise‐dominated landscapes without meta‐grid constraints or human‐in‐the‐loop interventions	[241]
Thermoelectric property optimization	Bayesian optimization with Gaussian process regression	Five‐dimensional parameter space: substrate temperature ($T_{\mathrm{sub}}$) and Knudsen cell temperatures T_Ga‐cell_, T_Sb‐cell_, T_As‐cell_, $+T_{\mathrm{Sb}-\mathrm{cell}}$, $T_{\mathrm{Sn}-\mathrm{cell}}$ controlling alloy composition and dopant concentration	Sn‐doped polycrystalline ${\mathrm{In}}_{1-x}{\mathrm{Ga}}_{x}{\mathrm{As}}_{1-y}{\mathrm{Sb}}_{y}$ (III–V thin films)	Direct III–V evidence	Lacks physical insight into the optimization process, necessitating secondary post‐analyses to elucidate the underlying physical mechanisms and trade‐offs	[242]
Battery anode compositional design and rate capability optimization	Bayesian optimization with Gaussian process regression	Knudsen cell temperatures for four elements ($T_{\mathrm{Ga}}$, $T_{\mathrm{In}}$, $T_{\mathrm{As}}$, $T_{\mathrm{Sb}}$)	Quaternary III–V thin films (${\mathrm{In}}_{1-y}{\mathrm{Ga}}_{y}{\mathrm{As}}_{1-x}{\mathrm{Sb}}_{x}$)	Transferable analogy	Does not directly predict the complex structural degradation mechanisms that govern long‐term cycling failure	[243]
Transmissive photocathode structure optimization and quantum efficiency prediction	3D numerical modeling and finite element method (COMSOL)	Geometric parameters (height, diameter), surface parameters (emission/recombination rates), external electric field, and Al composition	Transmissive ${\mathrm{Al}}_{x}{\mathrm{Ga}}_{1-x}N$ nanoarrays (III–V semiconductor)	Direct III–V evidence (applied to photoemission rather than PEC‐WS)	Lacks full systematic capabilities for predicting complex interactions such as surface states and thermal effects; operates as a deterministic solver rather than an autonomous machine‐learning framework	[244]

Soussou et al. directly applied AI/ML to III–V photoelectrochemical operation [239]. This work introduces an AI‐assisted ElectroPyroElectric (EPE) methodology to characterize an n‐type GaAs photoelectrode over 1 Hz–100 kHz. The complex impedance, obtained from 40 experimental frequency points, is up‐sampled and modeled by Support Vector Regression (SVR) to reconstruct a dense Nyquist diagram. Combining EPE with ML enables robust extraction of transport parameters and reveals low‐frequency capacitive behavior that is hidden in conventional impedance measurements. This combined EPE–SVR methodology provides a scalable and cost‐effective approach for characterizing GaAs‐based photoelectrodes and, more broadly, for improving the design of III–V semiconductor materials for solar hydrogen production.

A second category encompasses AI/ML approaches applied to III–V processing and characterization but not to PEC operation. For instance, Bayesian optimization has been employed to navigate the complex compositional space of quaternary InGaAsSb thin films for lithium‐ion battery anode applications, demonstrating the power of ML‐guided materials discovery for III–V systems. This machine‐learning‐guided compositional design is optimized for high‐rate battery performance rather than for water splitting but provides a blueprint for managing the complex structural degradation and phase transitions that similarly afflict PEC photoelectrodes. For instance, leveraging disorder‐driven optical phenomena such as multiple scattering resonances and light localization in photonic glass (PG) (GaAs) based photoelectrodes offers an alternative to conventional photonic crystals and inverse opals.

Similarly, neural network‐based frameworks have been developed to investigate the electronic properties of defective materials at finite temperatures, and multilevel perceptron emulation has been used to model optical absorption in PG‐supported TiO_2_/GaAs from finite‐difference time‐domain (FDTD) simulations. Anomaly detection in GaAs molecular structures using convolutional neural networks has also been demonstrated. The scientific goals of these combined approaches are twofold. First, to develop approaches to predictive models for high‐performance PG electrodes based on light absorption simulations and to fabricate, characterize, and optimize PG‐based photoelectrodes for solar‐to‐hydrogen and solar‐to‐fuel photoelectrochemical applications [245]. With this approach, PG‐based TiO_2_/GaAs photoelectrodes were designed to enhance PEC currents by up to 100 times those of equivalent ultra‐thin‐film photoanodes and several times those of the equivalent photonic crystal. The ML prediction of anomalies indirectly enhanced the PEC characteristics of the materials.

More fundamentally, predicting the electronic properties of defective materials at finite temperatures remains computationally expensive even with DFT due to the large number of atoms in the simulation cell and the multitude of thermally accessible configurations. Temperature directly influences the optoelectrical and electronic properties of III–V semiconductors in long‐term PEC‐WS under one sunlight. A neural network‐based framework has proven useful for efficiently investigating the electronic properties of defective semiconductors at finite temperatures [240]. Researchers developed two advanced equivariant graph neural networks: Message‐passing atomic cluster expansion (MACE) for atomic energies and forces and DeepH‐E3 (a method trained on DFT) for the electronic Hamiltonian (Figure 11ai–iv). Focusing on representative point defects in GaAs, they demonstrate computational accuracy comparable to DFT at a fraction of the computational cost, predicting the temperature‐dependent bandgap of defective GaAs directly from larger‐scale molecular dynamics trajectories with an accuracy of a few tens of meV. This study offers a unique way to replace conventional DFT analyses with a more cost‐effective AI tool and has the potential to be applied to other III–V semiconductor materials.

**FIGURE 11:**
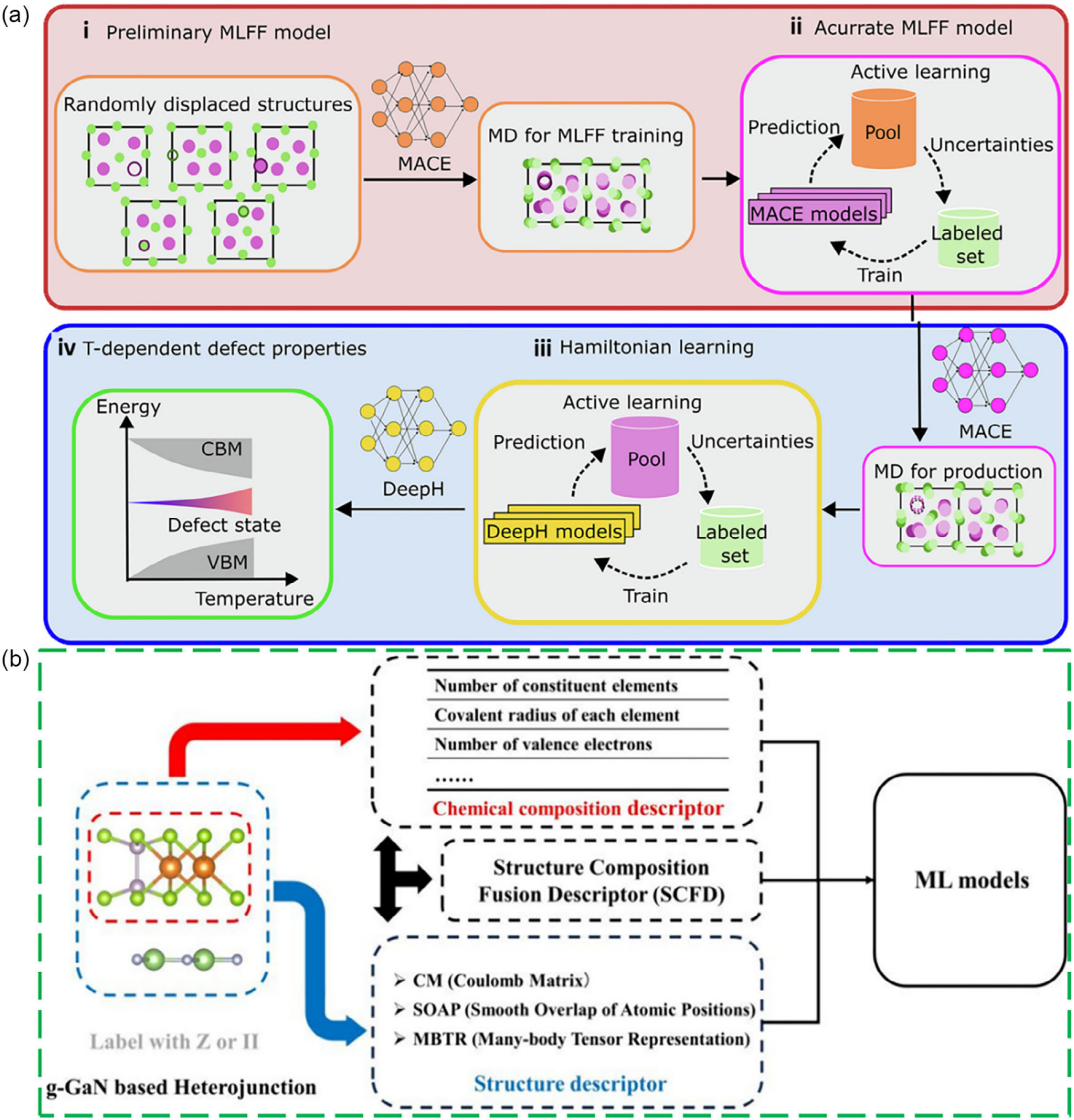
(a) (i) A preliminary MACE‐based machine‐learned force field (MLFF) is trained on randomly displaced defective structures and used to run short molecular dynamics (MD) simulations for further training. (ii) An accurate MLFF is obtained via active learning. MD‐generated configurations are stored in a pool set, where force uncertainties from an ensemble of MACE models are used to select samples for DFT labeling. These are added to the labeled set for iterative retraining. Longer MD simulations are run and enter the pool set of Hamiltonian learning. (iii) DeepH‐E3 models for Hamiltonian learning are also trained in an active learning scheme. Configurations in the pool set, which show high uncertainty in Hamiltonian matrix elements across an ensemble of DeepH models, are selected and added to the labeled set for iterative retraining. (iv) The final MLFF and Hamiltonian model are used together for predicting temperature‐dependent electronic properties of defects in semiconductors. Reproduced from ref. [240] under the terms of the CC BY license. (b) SCFD descriptor is mapped from the chemical composition descriptor of the material on the other side of the g‐GaN and the heterojunction structure descriptor and selected through machine learning models. Reproduced with permission from ref. [246] Copyright 2025, American Chemical Society.

A third category comprises studies on group III systems relevant to photocatalytic water splitting (PC‐WS). Leveraging a two‐dimensional materials database, a set of approximately 100 samples of II/Z‐type g‐GaN‐based van der Waals (vdW) heterojunctions was used to propose structure‐composition fusion descriptors (SCFD) [246]. Based on this, a binary ML model was developed to predict g‐GaN‐based II/Z‐type vdW heterojunctions (Figure 11b). The 10‐fold cross‐validation and test set accuracy reached 95%, demonstrating excellent robustness. The rationality of this model was further verified by applying first‐principles and nanoscale molecular dynamics (NAMD) theory. Based on the outcomes, a multisystem judgment method was successfully proposed for photocatalytic II/Z‐type heterojunctions with large bandgaps. Importantly, the provided case studies are still in their preliminary stages, and further efforts are needed to advance AI/ML‐derived control of III–V materials for PEC‐WS.

Finally, the literature reports several AI/ML supported photocorrosion prediction models for other photocatalytic systems and materials, yet a specific case directly demonstrating AI‐driven photocorrosion control of III–V photoelectrodes is still missing. To clarify this current landscape, Table 5 provides a comprehensive listing of various III–V material systems, dataset sizes, descriptors, and methodologies, explicitly distinguishing between direct III–V PEC evidence and transferable analogies. This distinction is critical to understand how AI/ML tools can be successfully transitioned from proxy models to advanced III–V PEC systems.

## 6 Summary, Existing Challenges, and Futuristic Overview

This review has presented an overview of highly promising strategies to enhance the PEC‐WS performance of III–V semiconductors, supported by relevant case studies and ML‐enhanced predictive models. Key strategies for inhibiting photocorrosion include surface passivation (acquired through physicochemical methods), crystal and facet control during growth, and the modification of bulk III–V materials with various other material classes, such as metallic and nonmetallic doping, as well as nanocomposite formation with materials such as ZnO and TiO_2_, among others. Each method is explained with a balanced number of examples, highlighting its respective advantages and disadvantages. Based on these findings, it can be concluded that integrating these strategies has the potential to produce industrially viable and durable III–V photoelectrodes for PEC‐WS in the future.

The epitaxial method is widely used to grow III–V semiconductors owing to its ability to achieve high growth yield and facet control. However, its high cost and procedural complexities often limit its scalability for commercial applications. As such, various methods should be utilized to grow the desired III–V semiconductors for PEC‐WS and other optoelectronic devices. To make sure III–V semiconductors last a long time, they need to undergo further research on passivation strategies to help make them durable. The selection of a passivation layer deposition technique and material should be based on overall cost, control, and efficiency. For instance, surface passivation using cost‐effective, nontoxic materials such as titanium and aluminum offers significant potential [46, 172, 247, 248]. The thickness of the passivation layer must be carefully optimized. It should be neither too thin to prevent effective photocorrosion control, nor too thick to hinder photon interaction at the photoelectrode interface [249]. Furthermore, employing multiple modifications on a single bulk electrode may introduce intrinsic and interfacial barriers, potentially degrading PEC performance rather than enhancing it. As described above, lattice mismatching remains a crucial growth issue in III–V semiconductors, leading to undesirable surface defects and detrimental dislocations [250, 251].

To bypass these experimental bottlenecks, lattice‐matched multijunction formation offers a promising alternative, avoiding the need for modification with foreign materials and enhancing photostability by orders of magnitude. PEC‐WS via multijunction semiconductors enables the direct conversion of photons into stored chemical energy in the form of hydrogen bonds. The multijunction electrode should be integrated by judiciously selecting semiconductors with optimal bandgaps. For instance, a state‐of‐the‐art p–n inverted metamorphic multijunction GaInP/GaInAs photocathode (IMM device) was grown in a custom‐built, ambient‐pressure metal‐organic vapor phase epitaxy reactor by the National Renewable Energy Laboratory (NREL) [52]. Figure 12a shows the structural diagrams of the parent and modified IMM devices. The GaInP/GaInAs photocathode, with a n‐AlInP window layer photocathode (IMM‐pnw), demonstrated reduced voltage losses of only 0.55 V compared with that of traditional p‐doped photocathodes and achieved 16% STH efficiency with maximum IPCE yield when using GaInP/GaInAs tandem absorbers (Figure 12b). The IMM devices with a back reflector significantly increase the photocurrent (white arrow). Replacing the 1.4‐eV GaAs bottom junction with a 1.2‐eV InGaAs layer enhances the photocurrent while reducing the photovoltage (orange arrows). Ultimately, optimizing the p–n GaInP doping profile results in a significantly higher voltage (blue arrow) compared with that of a uniformly doped p‐GaInP PEC top junction. As shown in Figure 12c, adding the n‐AlInP window layer and n‐GaInP passivation cap yields the greatest improvement in photocurrent and potential (green arrows). These findings demonstrate that careful selection of the multijunction arrangement using conventional materials can significantly advance PEC‐WS performance.

**FIGURE 12:**
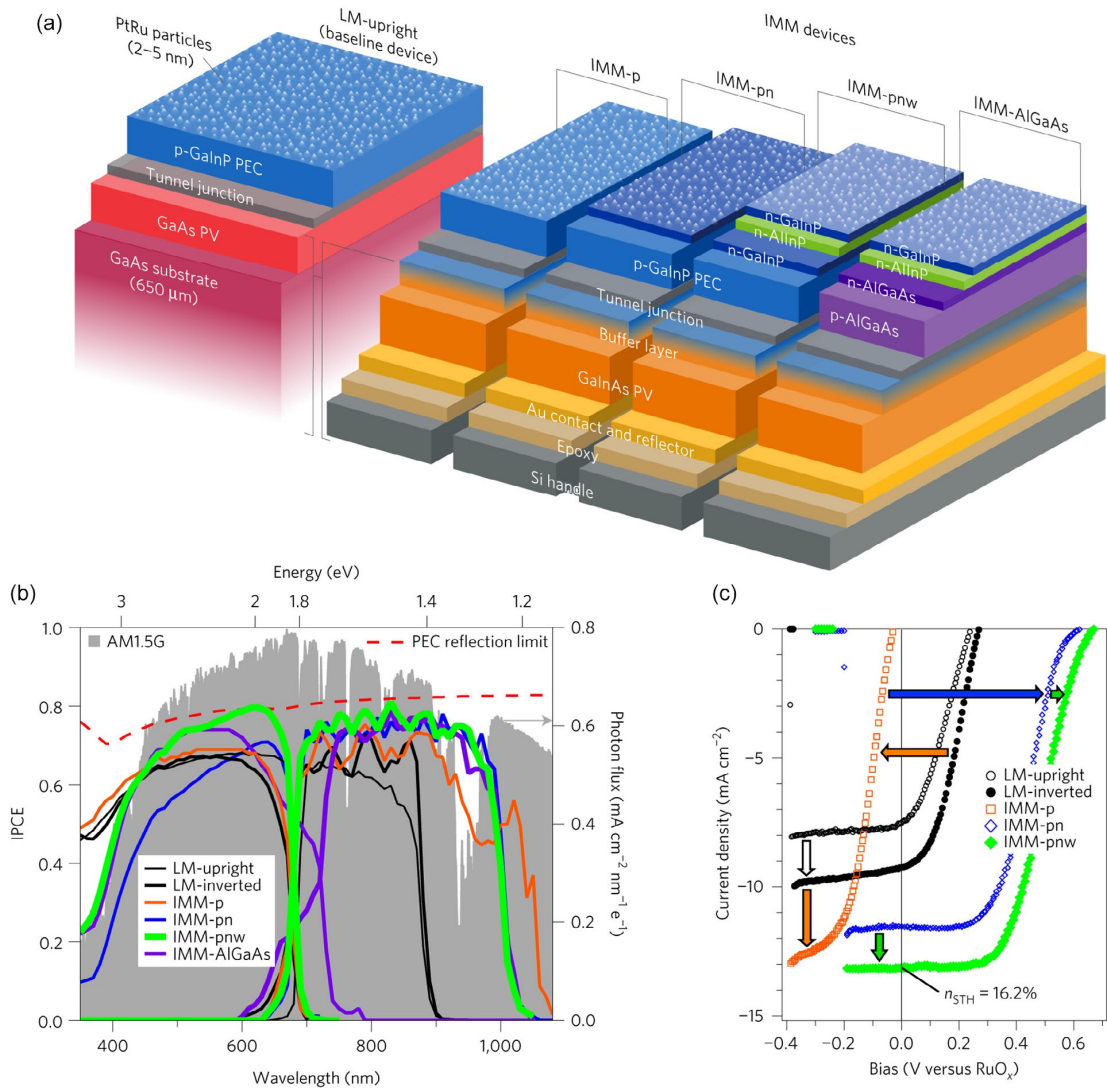
(a) Layer structure of the lattice‐matched (LM) upright GaInP/GaAs baseline device (left) and of four inverted metamorphic multijunction (IMM) devices in order of structural advancement (right). From left to right, IMM‐p replaces GaAs with lattice‐mismatched GaInAs, IMM‐pn adds an approximately 25 nm thin n‐GaInP layer, IMM‐pnw adds an n‐AlInP window layer capped by n‐GaInP, and IMM‐AlGaAs replaces the GaInP top junction with AlGaAs. (b) IPCE spectra of the devices overlaid on the one‐sun AM 1.5G reference spectrum (gray) with the PEC reflection limit (red dashed line). (c) Current density versus bias (V versus RuO_*x*_) under one‐sun illumination. The arrows indicate the gains from the back reflector (white), the lower bandgap GaInAs bottom junction (orange), the p–n GaInP top junction (blue), and the n‐AlInP window layer with n‐GaInP cap (green), which yields *η*_STH_ = 16.2%. Reproduced with permission from ref. [52]. Copyright 2017, Springer Nature.

However, discovering such intricate architectures through trial and error is highly inefficient. Therefore, the integration of ML and generative AI algorithms is imperative to predict the described issues well before experimental growth, thereby minimizing costs, labor, and material consumption. With the breakthrough in ML, generative AI, and computational tools, leveraging their advantages is essential when selecting appropriate III–V photoelectrodes for PEC‐WS studies. Various predictive models have been discussed in different sections of the review to underscore their importance for III–V‐based PEC‐WS devices. If used correctly, these predictive tools can help reveal previously unexplored traits of the frequently used GaAs, GaP, InGaP, and GaAsP photoelectrodes. The evaluation of these materials involves several parameters, such as optical properties (electronic structure and bandgap energies), thermodynamic attributes (Gibbs free energy calculations), electrostatic and van der Waals interactions, and electrolyte/electrode interface modeling [252]. The process typically begins with a comprehensive literature review to identify existing libraries and datasets of materials and datasets relevant to Group III–V semiconductors. The dataset includes specific information about the crystal structure, chemical composition, and electronic properties. This dataset serves as the foundation for identifying the most suitable materials for PEC‐WS applications. In the subsequent step, DFT‐based calculations are performed on the selected candidates to evaluate their optoelectronic and thermodynamic properties. The ranking of optoelectronic properties narrows down the number of suitable candidates. The information can be stored for future use, where it can be input into ML algorithms to analyze patterns and trends [253].

As a case study, Stafford et al. provided a computational/AI‐generated workflow for designing materials for water splitting using a sample of >30 000 undiscovered semiconductor compositions (Figure 13a). Their algorithm successfully generated suitable crystal structures with optimal light‐trapping, resistance to reduction/oxidation, and high efficiency in H_2_/O_2_ production. To achieve this, they used an ionic translation model trained on the Inorganic Crystal Structure Database [253]. These predictions were then screened for redox stability under HER and OER conditions before generating thermodynamically stable crystal structures and calculating accurate bandgap values for the compounds. This approach identifies numerous promising semiconductor candidates with ideal properties for artificial photosynthesis, offering a significant advance toward converting sunlight into chemical fuels. In a related study, Mondal et al. used machine learning models trained on first‐principles calculations to map the band‐gap magnitude and type (direct or indirect) in quaternary III–V materials, with a particular focus on the wide range of compositions and strains in GaAsPSb [237]. The ML models demonstrated significant accuracy in predicting band‐gap properties, producing composition‐strain‐band‐gap mappings or band‐gap phase diagrams (Figure 13b) for GaAsPSb. This diagram can be extended to other III–V materials for targeted applications. The ML model also predicts that, for strains within 5%, GaAsPSb compositions are not suitable for optoelectronic devices when intrinsic P concentrations are high, as this induces indirect bandgaps (Figure 13c). Similarly, ML‐assisted tools can streamline dopant screening and defect property prediction, potentially saving significant time and costs. By combining ML predictions with physical models in Technology Computer‐Aided Design simulations, researchers can effectively gather figures of merit and assess the potential impact of selected dopants on overall device performance [254].

**FIGURE 13:**
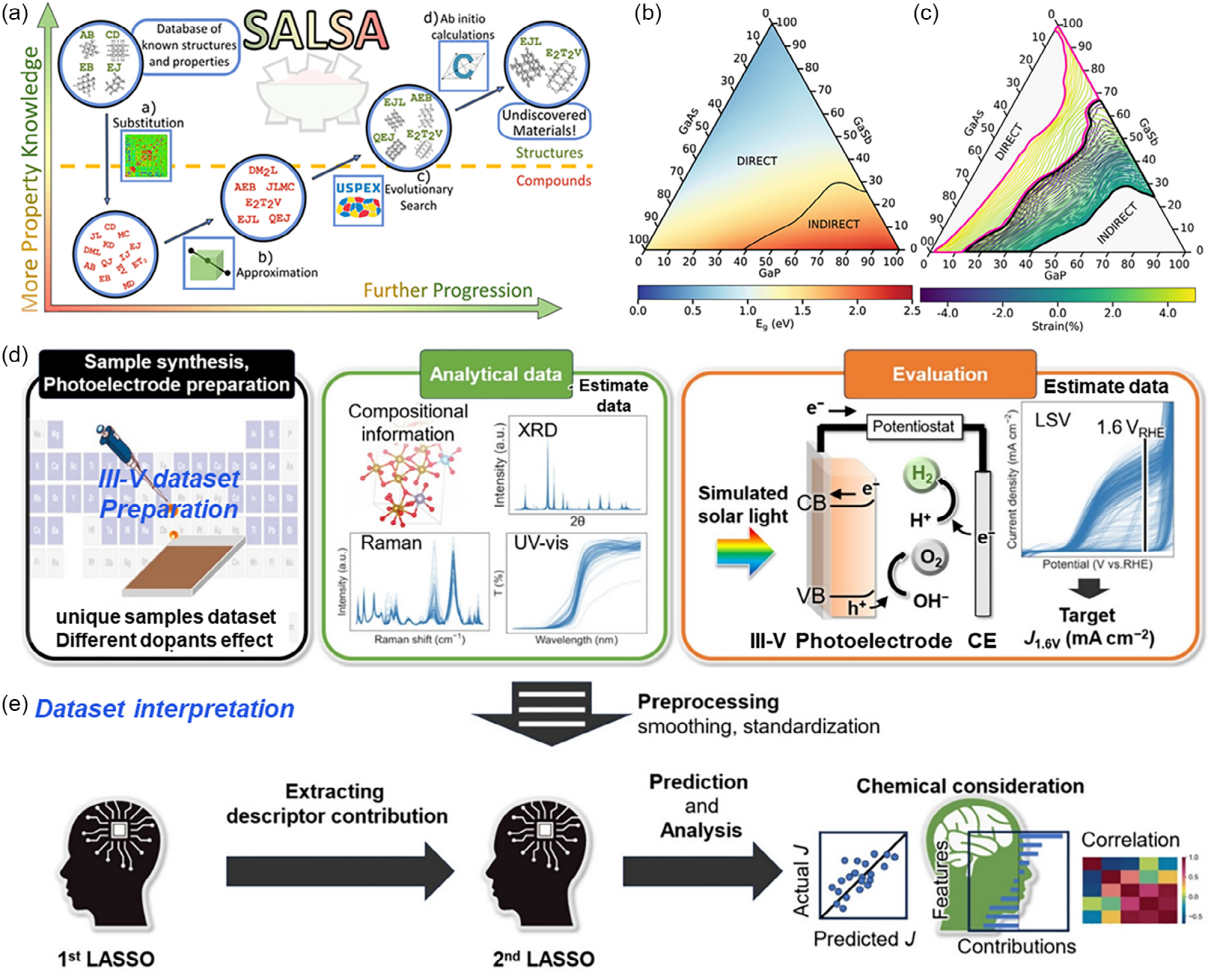
(a) The SALSA workflow for materials discovery. Known structures and properties from the Inorganic Crystal Structure Database (ICSD) are expanded by ionic substitution (step a) and approximation of new compositions (step b), followed by an evolutionary structure search with the USPEX algorithm (step c) and ab initio calculations with the CRYSTAL code (step d) to identify undiscovered materials. Reproduced with permission from ref. [253] Copyright 2023, AIP Publishing. (b,c) Machine‐learning bandgap phase diagrams of GaAsPSb. (b) Bandgap (*E*_g_) map at 0.0% strain, where the labels direct and indirect give the type of bandgap. (c) Bandgap map for strains up to 5.0%. The contour lines show the strain mapping, with the area under the magenta line corresponding to tensile strain and the bold black line marking compressive strain. Reproduced with permission from ref. [237]. Copyright 2023, AIP Publishing. (d,e) Proposed machine‐learning workflow for dopant screening in III–V photoelectrodes, adapted from the hematite workflow of [238] (d) Dataset preparation. Photoelectrodes with different dopants are synthesized, characterized by compositional analysis, XRD, Raman spectroscopy, and UV–vis spectroscopy, and evaluated by linear sweep voltammetry under simulated solar light, with the photocurrent density at 1.6 V_RHE_ (*J*_1.6 V_) as the target variable, as in the hematite study. (e) Dataset interpretation. Compositional information and analytical data are preprocessed (smoothing and standardization) and converted to elemental features with the XenonPy algorithm. A first LASSO regression extracts the descriptor contributions and a second LASSO regression performs the prediction. The selected descriptors and their correlations are then analyzed from a chemical perspective, and extrapolative predictions are used to screen dopant types and concentrations. Adapted with permission from ref. [238] Copyright 2025, American Chemical Society.

Nevertheless, some challenges remain, principally in accurately forecasting deep‐level capture rates and in addressing the lack of data on defect complexes and hexagonal‐phase semiconductors. For III–V semiconductors, the lack of comprehensive databases presents a significant challenge, highlighting the need for community‐wide adoption of FAIR (Findable, Accessible, Interoperable, and Reusable) data standards. In this regard, the ML‐enhanced experimental hybrid module can be a promising approach for predicting large datasets in similar PEC‐WS systems. For instance, regression models were developed to predict the PEC‐WS performance of multielement‐doped hematite photoelectrodes [238]. This model was based on elemental features derived from compositional information and analytical data (Figure 13d,e). The proposed two‐step LASSO method effectively eliminated descriptors with small regression coefficients, thereby improving generalization performance. While the present LASSO‐based approach successfully identifies dominant descriptors that contribute to photocurrent enhancement, it does not assume that all dopants act through the same physical or chemical mechanism. Rather, the results suggest that common underlying factors, partially consistent across dopants, can be extracted as interpretable descriptors, such as valence and ionic radius, even in chemically diverse multielement‐doping cases. The moderate prediction accuracy (*R* ^2^ = 0.76–0.81) also reflects the intrinsic complexity and dopant‐specific interactions in hematite photoelectrodes. Among the analytical data, Raman spectra were highly informative, allowing us to discuss, from a chemical perspective, the influence of semiconductor properties and vibrational characteristics on PEC performance. Finally, using all datasets, Nishimura et al. extrapolated the *J*_1.6 V_ of the virtual samples, identifying trends in elements likely to yield high‐ and low‐activity samples. Using advanced transfer learning paradigms, this hybrid model can be extended to virtually predict various III–V PEC‐WS systems by adopting the proposed scheme from Nishimura's work with some modifications.

The current literature includes computational and methodological approaches that could potentially be extended to photocorrosion prediction of III–V systems. However, there is a marked lack of direct demonstrations. The experts can learn AI concepts easily, and the ideas and results the poster produced were very interesting. To clarify, predicting states such as bandgaps, pristine states, dopant effects, etc., is different from predicting the photocorrosion effect. The challenge is not just a technical one, it is also a conceptual one. Photocorrosion is a non‐equilibrium, multi‐scale dynamic process that couples changes in internal electronic structure with catastrophic mechanical and chemical evolution, all occurring on short timescales. Such processes are difficult to model using the static, thermodynamic, composition‐centric descriptors such as bandgap, defect formation energy, oxidation energy, etc., used by many current ML approaches. Heterogeneity is exhibited in the dynamic corrosion interfaces, thereby causing static crystal databases used to train models to have significant uncertainty and a high failure rate on out‐of‐distribution predictions.

To establish relevant degradation targets, future AI models should incorporate anodic dissolution rates, defect‐generation energy, and oxide‐leaching kinetics. Future work should focus on the PEC‐specific datasets for III–V materials, time‐dependent and operational descriptors, and in‐situ characterization and ML to enable real‐time feedback on corrosion events. The community should be able to distinguish and appropriately apply different computational methods to problems of insufficient data. An example of first principles‐DFT thermodynamic studies for atomistics offers surrogate ML models for fast screening, uses Generative AI for suggesting novel passivation topology, and finally Bayesian Optimization for navigating and enabling customized multiparameter experimental synthesis landscape. Finally, autonomous experimentation will be critical for linking theory and reality in the future. The hypothesis of “AI‐guided photocorrosion inhibition” of III–V semiconductors must be an aspiration until such studies appear. The fact that there is an inadequate database at present is more indicative of the field's early developmental stage and serves as an appeal.

In summary, the outstanding PEC‐WS outcomes of III–V devices in terms of high efficiency (STH, IPCE, QE) are considerable positives, yet high costs, stability challenges, and complex synthesis are issues that remain to be solved. While ML and AI have accelerated the empirical optimization of both intrinsic and extrinsic modifications for III–V materials, their current deployment often prioritizes predictive accuracy over mechanistic understanding. The field's future hinges not on abandoning data‐driven methods but on constraining them with physically and chemically informed descriptors, such as surface bond coordination, band‐edge positions, and defect formation energies, derived from analytical tools and first‐principles calculations.

Finally, claims regarding the immediate industrial viability of AI‐designed III–V photoelectrodes must be moderated. Given the exceptionally high cost of III–V epitaxial growth compared with silicon, the application of predictive AI frameworks, along with rigorous techno‐economic analysis (TEA) and life cycle assessment (LCA) algorithms, will be pivotal to establishing the genuine commercial viability of such electrodes prior to laboratory synthesis. These high manufacturing costs must be clearly justified by massive improvements in long‐term durability, scale‐up feasibility, and overall reductions in solar‐to‐hydrogen lifecycle emissions. The ultimate game‐changer will be the transition of AI from predictions only through pre‐synthetic screening to real‐time, operando insights into degradation dynamics through machine‐vision‐integrated spectroscopy. Hybrid frameworks that marry ML pattern recognition with experimental datasets and economic modeling offer the most robust pathway for prospective investigations toward industrially scaled, highly durable artificial photosynthesis.

## Funding

This research was supported by the Chung‐Ang University Young Scientist Scholarship in 2021. The work is also supported by the Brain Pool program funded by the Ministry of Science and ICT through the National Research Foundation of Korea (No. NRF‐2021H1D3A2A02044205) and a grant funded by the Korean government (MSIT) (No. RS‐2023‐00277937).

## Conflicts of Interest

The authors declare no conflicts of interest.

## Data Availability

The data that support the findings of this study are available from the corresponding author upon reasonable request.
